# Percutaneous and surgical tracheostomy in critically ill adult patients: a meta-analysis

**DOI:** 10.1186/s13054-014-0544-7

**Published:** 2014-12-19

**Authors:** Christian Putensen, Nils Theuerkauf, Ulf Guenther, Maria Vargas, Paolo Pelosi

**Affiliations:** Department of Anesthesiology and Intensive Care Medicine, University of Bonn, Sigmund-Freud-Straße 25, 53105 Bonn, Germany; Department of Surgical Sciences and Integrated Diagnostics, University of Genoa, Largo Rosanna Benzi 8, 16132 Geneva, Italy

## Abstract

**Introduction:**

The aim of this study was to conduct a meta-analysis to determine whether percutaneous tracheostomy (PT) techniques are advantageous over surgical tracheostomy (ST), and if one PT technique is superior to the others.

**Methods:**

Computerized databases (1966 to 2013) were searched for randomized controlled trials (RCTs) reporting complications as predefined endpoints and comparing PT and ST and among the different PT techniques in mechanically ventilated adult critically ill patients. Odds ratios (OR) and mean differences (MD) with 95% confidence interval (CI), and I^2^ values were estimated.

**Results:**

Fourteen RCTs tested PT techniques versus ST in 973 patients. PT techniques were performed faster (MD, −13.06 minutes (95% CI, −19.37 to −6.76 (*P* <0.0001)); I^2^ = 97% (*P* <0.00001)) and reduced odds for stoma inflammation (OR, 0.38 (95% CI, 0.19 to 0.76 (*P* = 0.006)); I^2^ = 2% (*P* = 0.36)), and infection (OR, 0.22 (95% CI, 0.11 to 0.41 (*P* <0.00001)); I^2^ = 0% (*P* = 0.54)), but increased odds for procedural technical difficulties (OR, 4.58 (95% CI, 2.21 to 9.47 (*P* <0.0001)); I^2^ = 0% (*P* = 0.63)). PT techniques reduced odds for postprocedural major bleeding (OR, 0.39 (95% CI, 0.15 to 0.97 (*P* = 0.04)); I^2^ = 0% (*P* = 0.69)), but not when a single RCT using translaryngeal tracheostomy was excluded (OR, 0.58 (95% CI, 0.21 to 1.63 (*P* = 0.30)); I^2^ = 0% (*P* = 0.89)). Eight RCTs compared different PT techniques in 700 patients. Multiple (MDT) and single step (SSDT) dilatator techniques are associated with the lowest odds for difficult dilatation or cannula insertion (OR, 0.30 (95% CI, 0.12 to 0.80 (*P* = 0.02)); I^2^ = 56% (*P* = 0.03)) and major intraprocedural bleeding (OR, 0.29 (95% CI, 0.10 to 0.85 (*P* = 0.02)); I^2^ = 0% (*P* = 0.72)), compared to the guide wire dilatation forceps technique.

**Conclusion:**

In critically ill adult patients, PT techniques can be performed faster and reduce stoma inflammation and infection but are associated with increased technical difficulties when compared to ST. Among PT techniques, MDT and SSDT were associated with the lowest intraprocedural risks and seem to be preferable.

**Electronic supplementary material:**

The online version of this article (doi:10.1186/s13054-014-0544-7) contains supplementary material, which is available to authorized users.

## Introduction

Tracheostomy is among the most commonly conducted procedures in critically ill patients. Despite percutaneous tracheostomy (PT) techniques gaining acceptance, the debate continues about their precise indications, their possible advantages over conventional surgical tracheostomy (ST), and whether one PT technique is superior to the others [1–3]. Observational studies indicate that ST is still performed in 33 to 50% of critically ill patients [4,5], especially in the presence of neurological disorders.

Conflicting results have been reported in three previous meta-analyses comparing complication rates between PT techniques and ST [6–8]. All of these meta-analyses included only multiple dilator tracheostomy (MDT) [9], guide wire dilating forceps (GWDF) [10], and translaryngeal tracheostomy (TLT) [11] in the PT group. Since newer PT techniques such as single-step dilation tracheostomy (SSDT) [12], rotational dilation tracheostomy (RDT) [13], or balloon dilation tracheostomy (BDT) [14] are increasingly used due to easy application and shorter procedure times [15], previous meta-analyses may not reflect current clinical practice. Recently, a meta-analysis including randomized, controlled trials (RCTs) comparing different PT techniques concluded that SSDT appears to be superior in terms of safety and success rate [15].

Our objective was to determine whether a specific PT technique is superior to ST or to other PT techniques in adult critically ill patients with an indication for tracheostomy with respect to complications during the procedure (major and minor bleeding, technical difficulties, false route, subcutaneous emphysema, pneumothorax, and oxygen desaturation) or after the procedure (major and minor bleeding, stoma inflammation or infection, tracheomalacia, and tracheal stenosis), the length of the procedure and hospital survival.

## Materials and methods

### Data sources and searches

We aimed to identify all RCTs assessing the complications and outcomes between PT and ST and among the different PT techniques in adult critically ill patients. The electronic search strategy applied standard filters for identification of RCTs. Databases searched were the Cochrane Central Register of Controlled Trials (CENTRAL, The Cochrane Library Issue 3, 2012), MEDLINE (from inception to July 2013), and EMBASE (from inception to July 2013). We did not apply language restrictions. Our search included the following key words: tracheotomy, tracheostomy, percutaneous, dilatation, surgical, Griggs, forceps, Percutwist, Ciaglia, Blue Rhino, Fantoni, translaryngeal, Blue Dolphin, multiple dilator technique, guide wire dilating forceps, translaryngeal technique, single-step dilation technique, rotational dilation technique balloon dilation technique, critical care, intensive care, critically ill, and random. In addition to the electronic search, we checked cross-references from original articles and reviews. We retrieved additional studies by hand searching the abstracts of the meetings of the American Thoracic Society, the Society of Critical Care Medicine, and the European Society of Intensive Care Medicine held from 2010 to 2013. Completed but unpublished studies were identified by searching the websites for the Public Registers of Clinical Trials [16,17]. Neither ethical approval nor patient consent was needed in this meta-analysis.

### Selection of studies

We restricted the analysis to RCTs to guarantee control of selection bias. Study designs containing cointerventions unequally applied to the treatment and control group as well as nonrandomized or crossover trials were not included.

RCTs reporting complications as predefined endpoints and comparing PT with ST and comparing the different PT techniques in mechanically ventilated adult critically ill patients were considered for inclusion.

PT had to be performed according to MDT, GWDF, TLT, SSDT, RDT, or BDT. A description of the different PT techniques and synonyms are presented in Additional file 1. PT and ST techniques had to be performed either in the ICU or in the operating room. Studies of tracheotomy in emergency airway management, in infants and children, in not critically ill or homecare patients and those published only as a letter were excluded. Authors were contacted to clarify details of trials, if necessary.

### Outcome measures

The primary outcomes were complications during and after the procedure. Complications during the procedure included major and minor bleeding, technical difficulties, false route, subcutaneous emphysema, pneumothorax, and oxygen desaturation. Complications after the procedure included major and minor bleeding, stoma inflammation or infection, tracheomalacia, and tracheal stenosis. Definitions of complications are presented in Table 1.

**Table 1:** **Definitions of complications**

**Complication**	**Description**
Minor postprocedural bleeding	
Cuff leak	Perforation of tracheostomy tube balloon within the first 24 hours
Difficult dilatation	Need for excessive force and/or enlargement of the incision, and/or more than three passes of the largest dilator
Difficult insertion	Need for more than two passes of tracheostomy tube/dilator combination before successful insertion
Esophageal perforation	Esophageal insertion of needle and/or guide wire and/or tracheostomy tube
False route	Paratracheal insertion of tracheostomy tube
Gastric aspiration	Tracheal aspiration of gastric contents during the procedure
Hypotension	Systolic blood pressure lower than 90 mmHg
Hypoxemia	Pulse oximetry arterial oxygen saturation lower than 90%
Inflammation	Edema and/or erythema and/or tenderness, no pus of the stoma
Infection	Signs of inflammation and (culture-positive) purulent discharge (requiring antibiotic therapy) of the stoma
Minor bleeding	Bleeding controlled by compression or insertion of the tracheotomy tube, need for dressing change, estimated external blood loss <20 ml
Major bleeding	Need for surgical exploration, suture ligation, electrocautery and/or transfusion of packed red blood cells, estimated blood loss of either >7 gauze swabs or >20 ml external or intratracheal blood loss
Loss of airway	Absence of airway access, requiring reintubation
Pneumothorax	Intrapleural air on postoperative chest radiograph
Pneumomediastinum	Mediastinal air on postoperative chest radiograph
Subcutaneous emphysema	Subcutaneous air on postoperative chest radiograph
Tracheo-innominate fistula	Erosion of the innominate vein through tracheostomy tube

Secondary outcomes included the length of the procedure and hospital survival.

### Data extraction and quality assessment

Initial selection was performed by screening titles and abstracts by two pairs of independent reviewers (NT and UG, PP and CP). Citations were screened by each reviewer and selected for further evaluation if they reported in a RCT comparison between PT and ST or among the different PT techniques in adult critically ill patients or if the title or abstract did not give enough information to make an assessment. For detailed evaluation, a full-text copy of all studies of possible relevance was obtained and data from each study were again extracted independently by paired reviewers (NT and UG, PP and CP), using a prestandardized data abstraction form. One pair of reviewers (NT and UG) was not informed about authors, journal, institutional affiliation, and date of publication. Data extracted from the publications were checked by a further reviewer (UG) for accuracy. Quality assessment of these studies included: use of randomization; reporting of allocation concealment; blinding; adequate selection and description of study population with respect to inclusion and exclusion criteria; comparability of the groups at baseline; use of a predefined treatment protocol; absence of confounders; absence of cointerventions; *a priori* definition of primary and secondary outcome parameters; use of intention-to-treat analysis; extent of follow-up; *a priori* calculation of sample size; patients screened and included in the trial; reports on patients lost to follow-up; and planned or premature termination of the RCT. Two reviewers (NT and UG) independently used these criteria to abstract trial quality. We resolved any disagreements by consensus in consultation with a third reviewer (PP) if needed.

### Risk of bias

To evaluate potential publication bias, a weighted linear regression was used, with the natural log of the odds ratio as the dependent variable and the inverse of the total sample size as the independent variable. This is a modified Macaskill’s test, which gives more balanced type I error rates in the tail probability areas compared with other publication bias tests [18].

### Data synthesis and analysis

We classified the following comparisons: pooled PT techniques versus ST, MDT versus ST, pooled MDT + SSDT versus pooled GWDF + RDT + BDT, and pooled MDT + SSDT versus GWDF.

### Qualitative analysis

A narrative summary approach was used to explore study characteristics and quality indicators to analyze study-to-study variations and their implications for the outcomes reported in the included RCTs [19,20].

### Quantitative analysis

The meta-analysis was performed according to the Cochrane Collaboration guidelines [21]. All statistical analyses were performed with Review Manager (Revman, The Cochrane. Collaboration, Oxford, UK), software for preparing and maintaining Cochrane systematic reviews [21]. The pooled effects estimates for binary variables were expressed as odds ratios with 95% confidence interval (CI), whereas continuous variables were expressed as mean differences with 95% CI. We tested the difference in estimates of treatment effect between the treatment and control groups for each hypothesis using a two-sided *z* test with statistical significance considered at *P* <0.05.

We examined heterogeneity using the Cochran *Q* test and the *I*^2^ test [22,23]. We predefined heterogeneity as low, moderate, or high with *I*^2^ values above 25%, 50%, or 75% respectively [23]. Meta-analysis with a random-effects model was applied with *I*^2^ values above 25% [24]. Otherwise, we performed the meta-analysis using a fixed-effect model. However, the possibility of a type II (false negative) error must be considered and a thorough attempt was made to identify clinical heterogeneity or sources of bias. We considered one-tailed *P* <0.05 as significant.

Interobserver agreement on selection of articles for inclusion and quality assessment was measured with Cohen’s (unweighted) κ statistic [25]. We considered a κ value of greater than 0.8 to indicate acceptable agreement.

## Results

### Study selection

**Figure 1:**
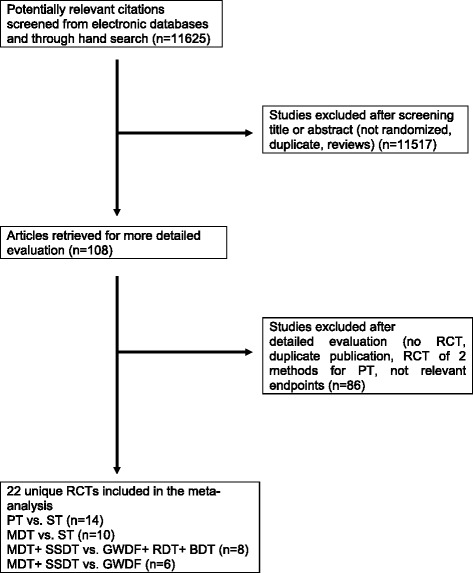
**Literature search and selection.** BDT, balloon dilatation tracheotomy; GWDF, guide wire dilatation forceps; MDT, multiple dilatation tracheotomy; *n*, number; PT, percutaneous tracheotomy; RCT, randomized controlled trial; RDT, rotational dilatation tracheotomy; SSDT, single-step dilatation tracheotomy; ST, surgical tracheotomy.

Our initial electronic and manual search identified 11,625 studies. Of these, we excluded 11,517 articles after screening the title or abstract. We retrieved 108 studies for more detailed analysis, and excluded 86 of these because they were not RCTs, they did not evaluate PT or ST in critically ill patients, they were duplicated references, or they were not relevant (Figure 1). The two reviewer teams completely agreed (κ = 1) on the selection of included studies.

### Study description

Table 2 summarizes the study characteristics and quality scores. Although all 22 studies [26–47] were published in English, they represent international experience, including data from 14 countries. All but one RCT [46] were conducted in a single center.

**Table 2:** **Quality of included studies**

**Study**	**Random assignment**	**Allocation concealment**	**Blinding**	**Adequate selection and description of study population**	**Comparability of groups**	**Pre-defined treatment protocol**	**Absence of confounders**	**Absence of cointerventions**	***A priori*** **definition of outcome**	**ITT**	**Power analysis**	**Follow-up duration**	**Patients screened/included in trial**	**Reports on patients lost to follow-up**	**Planned or premature termination of study**
**Studies comparing PT vs. ST (pooled PT vs. ST, including MDT vs. ST)**															
Hazard and colleagues [36]	Yes	Unclear	No	Yes	Yes, no statistical differences in age, gender, duration of intubation, underlying diagnosis, APACHE II score and coagulation parameters	Yes	Yes	Yes	Yes	Yes	No	12 weeks after decannulation	NR/ 46	No	As planned
Crofts and colleagues [31]	Yes	No, randomization by alternating weeks	No	Yes	Yes, no statistical differences in age, gender, duration of intubation and APACHE II score	Yes	Yes	Yes	Yes	Yes	No	3 months	NR/ 53	No	As planned
Friedman and colleagues [34]	Yes	Unclear, randomization by random number tables	No	Yes	Yes, no statistical differences in age, gender, duration of intubation, underlying diagnosis, APACHE II score and coagulation parameters	Yes	Yes	Yes	Yes	Yes	No	NR	NR/ 53	No	As planned
Holdgaard and colleagues [38]	Yes	Unclear	No	Yes	Yes, no statistical differences in age, gender and duration of intubation	Yes	Yes	Yes	Yes	Yes	No	NR	NR/ 60	No	As planned
Gysin and colleagues [35]	Yes	Unclear, randomization by computer-generated random list	No	No	Yes, no statistical differences in age, gender and duration of intubation	Yes	Yes	Yes	Yes	Yes	No	3 months after decannulation	NR/ 70	Yes, 40 lost	As planned
Porter and Ivatury [42]	Yes	Unclear, randomization by sealed envelopes	No	No	Yes, no statistical differences in age, gender and duration of intubation	Yes	Yes	Yes	Yes	Yes	No	NR	NR/ 70	No	As planned
Heikkinen and colleagues [37]	Yes	No, randomization by the lot	No	No	Yes, no statistical differences in age, gender and duration of intubation	Yes	Yes	Yes	Yes	Yes	No	18 months	NR/ 57	Yes, 46 lost	As planned
Freeman and colleagues [33]	Yes	Unclear	No	Yes	Yes, no statistical differences in age, gender, duration of intubation, saps and coagulation parameters	Yes	Yes	Yes	Yes	Yes	No	Hospital discharge	NR/ 80	No	As planned (hospital discharge)
Melloni and colleagues [40]	Yes	Unclear	No	Yes	Yes, no statistical differences in age, gender, duration of intubation and SAPS II	Yes	Yes	Yes	Yes	Yes	No	6 months	NR/ 50	Yes, 32 lost	As planned (6 months)
Sustic and colleagues [44]	Yes	Unclear	No	Yes	Yes, no statistical differences in age and gender	Yes	Yes	Yes	Yes	Yes	No	ICU discharge	NR/ 16	No	As planned (ICU discharge)
Wu and colleagues [47]	Yes	Unclear, randomization by computer-generated random list	No	No	Yes, no statistical differences in age, gender, duration of intubation and underlying diagnosis	Yes	Yes	Yes	Yes	Yes	No	2 years	NR/ 83	Yes, 52 lost	As planned
Antonelli and colleagues [28]	Yes	Unclear, randomization by computer-generated random list	No	Yes	Yes, no statistical differences in age, gender, duration of intubation, underlying diagnosis and SAPS II	Yes	Yes	Yes	Yes	Yes	Yes	360d	825/ 139	Yes, 36 lost	As planned (12 months)
Tabaee and colleagues [45]	Yes	No, randomization by the last number of the medical record	No	No	Yes no statistical differences in age, gender and duration of intubation	Yes	Yes	Yes	Yes	Yes	No	1 week	45/43	No	As planned (1 week)
Silvester and colleagues [43]	Yes	Yes, by sealed envelopes	No	Yes	Yes, no statistical differences in age, gender, duration of intubation, underlying diagnosis, APACHE II score and coagulation parameters	Yes	Yes	Yes	Yes	Yes	Yes	15 to 40 months	298/ 200	Yes, 119 lost	Terminated early after 200 patients due to lack of power
**Studies comparing different PT techniques (pooled MDT/SSDT vs. pooled GWDF/ RDT/BDT, including MDT/SSDT vs. GWDF alone)**															
Nates and colleagues [41]	Yes	Yes, randomization by sealed envelopes	No	Yes	Yes, no statistical differences in age, gender, duration of intubation, APACHE II score and coagulation parameters	Yes	Yes	Yes	Yes	Yes	No	Day 7	NR/ 100	Yes, 8 lost	As planned (day 7)
van Heurn and colleagues [46]	Yes	Unclear	No	No	Yes, no statistical differences in age, gender and duration of intubation	Yes	Yes	Yes	Yes	Yes	No	ICU discharge	NR/ 127	No	As planned (ICU discharge)
Ambesh and colleagues [26]	Yes	Unclear	No	Yes	Yes, no statistical differences in age, gender, duration of intubation, APACHE II score and BMI	Yes	Yes	Yes	Yes	Yes	No	8 weeks after decannulation	NR/ 60	Yes, 27 lost	As planned
Byhahn and colleagues [29]	Yes	Unclear, randomization by computer-generated random list	No	No	Yes, no statistical differences in age, gender, duration of intubation and BMI	Yes	Yes	Yes	Yes	Yes	No	NR	NR/ 70	No	As planned
Anon and colleagues [27]	Yes	Unclear, randomization by number block procedure	No	Yes	Yes, no statistical differences in age, gender, duration of intubation and APACHE II score	Yes	Yes	Yes	Yes	Yes	No	Day 282 ± 198	NR/ 53	Yes, 36 lost	As planned
Kaiser and colleagues [39]	Yes	Yes, randomization by sealed envelopes	No	Yes	Yes, statistically proven for age, gender, duration of intubation and SAPS II	Yes	Yes	Yes	Yes	Yes	No	NR	NR/ 100	No	As planned
Cianchi and colleagues [30]	Yes	Unclear, randomization by computer-generated random list	No	Yes	Yes, no statistical differences in age, gender, duration of intubation BMI and coagulation parameters	Yes	Yes	Yes	Yes	Yes	No	Hospital discharge	78/ 70	Yes, 0 lost	As planned
Fikkers and colleagues [32]	Yes	Yes, randomization by sealed envelopes	No	Yes	Yes, no statistical differences in age, gender, duration of intubation, SOFA score and BMI	Yes	Yes	Yes	Yes	Yes	No	3 months after decannulation	145/ 120	Yes, 73 lost	As planned

When adequate selection and description of the study population was classified as ‘no’, enough information was provided to ensure the patients met the criteria for inclusion in the systematic review (that is, critically ill, in an ICU, and so forth). APACHE, Acute Physiology and Chronic Health Evaluation; BDT, balloon dilatation tracheotomy; BMI, body mass index; GWDF, guide wire dilatation forceps; MDT, multiple dilatation tracheotomy; NR, not reported; PT, percutaneous tracheotomy; RDT, rotational dilatation tracheotomy; SAPS, Simplified Acute Physiology Score; SOFA, Sequential Organ Failure Assessment; SSDT, single step dilatation tracheotomy; ST, surgical tracheotomy; ITT, intention to treat.

We identified the definition of the patient population and severity of critical illness, exclusion criteria, tracheostomy techniques, the medical specialty performing the tracheotomy, the location of performance, use of bronchoscopy, and reported complications of the trials as key sources of between-study variation. Qualitative analysis of key study characteristics and quality indicators revealed the following differences.

#### Patient population and severity of critical illness

A mixed population of critically ill patients was investigated in 16 RCTs [27–31,33,34,36,38–43,45,47], trauma patients in one RCT [44], and surgical patients in one RCT [37]. The patient population was not described in four RCTs [26,32,35,46]. Seven RCTs reported the Acute Physiology and Chronic Health Evaluation Score [26,27,31,33,34,41,43], one RCT reported the Sequential Organ Failure Assessment Score [32], and four RCTs reported the Simplified Acute Physiology Score II [28,36,39,40].

#### Exclusion criteria

Fourteen RCTs reported coagulation disorders [27,28,30–34,36,39,41–44,46], 12 RCTs reported difficult anatomy of the neck [26–28,32–34,36,38,39,41,43,47], 14 RCTs reported neck pathology [27,28,30,32,34,36–41,43,46,47], six RCTs reported spine pathology [28,29,31,39,43,46], 11 RCTs reported pathology of the thyroid gland [27,28,30–32,34,36,37,40,41,43], three RCTs reported emergency tracheotomy [28,41,42], two RCTs reported difficult airways [29,33], nine RCTs reported previous tracheotomy [28,30,34–38,40,43], four RCTs reported need for high airway pressures and/or high inspiratory oxygen concentrations [27,32,34,46], and two RCTs reported increased intracranial pressure as exclusion criteria [30,32].

#### Tracheostomy techniques

ST was compared with MDT in 10 RCTs [31,33–36,38,40,42,43,47], with SSDT in one RCT [45], with TLT in one RCT [28], and with GWDF in two RCTs [37,44]. MDT was compared with GWDF in three RCTs [39,41,46]. GWDF was compared with SSDT in three RCTs [26,27,32], and SSDT was compared with RDT in one RCT [28] and with BDT in one RCT [30] (Additional file 2).

#### Medical specialty performing the tracheotomy

STs were performed by surgeons in 10 RCTs [31,34,36–38,40,42–44,47], by ear, nose, and throat physicians in three RCTs [28,35,45], and was not specified in one RCT [33]. PTs were performed by intensivists in 12 RCTS [26–30,32,34,39–41,43,44], by surgeons in four RCTs [37,42,46,47], by ear, nose, and throat physicians in three RCTs [31,35,45], and was not specified in three RCTs [33,36,38]. Tracheostomy was performed by the same medical specialty in 12 RCTs (intensivists in seven RCTs, surgeons in four RCTs, or ear, nose and throat specialists in one RCT) and by different specialties in six RCTs (intensivists and surgeons in four RCTs, ear, nose and throat specialists and surgeons in one RCT, and intensivists and surgeons in one RCT, respectively).

#### Location of procedure

STs were performed in the operating room in seven RCTs [28,31,33,34,38,44,47], in the ICU in three RCTs [37,43,45], and in the ICU or the operating room in four RCTs [35,36,40,42]. In all but one RCT [38], PTs were performed in the ICU [26–37,39–47]. In six out of the 14 RCTs comparing PT with ST, PT was performed in the ICU while ST was performed in the operating room. Both procedures were performed in the ICU in four out of 14 RCTs, while ST was performed in the ICU or in the operating room in three RCTs. In one study, PT and ST were performed in the operating room.

#### Use of bronchoscopy

Bronchoscopy was regularly used in nine RCTs [29,30,32,33,35,39,40,42,45], not used in 11 RCTs [26–28,31,34,36–38,41,44,46], and only used if complications occurred in two RCTs [43,47].

#### Reported complications

A summary of the definition of complications used in the included RCTs is presented in Table 1. Reported complications were defined *a priori* and systematically assessed in all included RCTs. Bleeding during tracheostomy was reported in 22 RCTs and after intervention in 17 out of 22 RCTs [26,28,30,32–38,40–45,47]. Thirteen RCTs used definitions to distinguish between minor and major bleeding complications [26–28,31,32,34,36,38,41–43,45,46]. Inflammation of the stoma was assessed in six RCTs [28,29,38,42–44] and infection of the stoma in 17 RCTs [26–29,31,32,34–38,40–44,47]. One RCT reported the use of microbiologic data to verify infection [28]. Nineteen RCTs reported technical difficulties [26,27,29–33,35–43,45–47]. Difficult insertion was reported in six RCTs [29,32,33,35,45,47], difficult dilatation in 14 RCTs, and difficult insertion and dilatation in four RCTs [26,27,29–32,35–38,40,42,45,46], and difficult insertion and dilatation in four RCTs [29,32,35,45]. Three RCTs did not specify the technical difficulties [39,41,43]. During the procedure a false route was reported in 12 RCTs [27,29,30,32,34,35,38,40,41,43,45,46], subcutaneous ememphysema in nine RCTs [26–29,31,34,35,38,39], pneumothorax in 12 RCTs [26,27,30–32,35,36,38,39,41–43], desaturation in 14 RCTs [26–30,32,34,35,38,39,42–45], and hypercapnia in three RCTs [26,43,44] CTs did not specify the technical difficulties [39,41,43]. Three RCTs investigated tracheal malacia [28,40,47] and nine RCTs investigated tracheal stenosis [27,28,30,32,35,36,40,43,47] after tracheotomy. In all included studies, there was great accordance regarding the classification of stomal inflammation and infection, technical difficulties differentiating difficult dilatation from difficult insertion and for major and minor bleeding complications, respectively. Regarding tracheal stenosis and tracheomalacia, patients in all but one RCT reporting on these late complications were assessed by means of an *a priori* defined and planned and clinical, radiological (by means of conventional tomography or MRI) or endoscopic examination.

### Evidence synthesis

#### Percutaneous tracheostomy techniques versus surgical tracheostomy

Analysis of pooled PT techniques versus ST included a total of 973 patients in 14 RCTs. The only study characteristics that may explain differences in the complication rates were different PT techniques. One RCT using TLT carries 39% of the weight in the pooled effect and significantly accounts for the positive effect of PT on the risk for major postprocedural bleeding [28].

**Figure 2:**
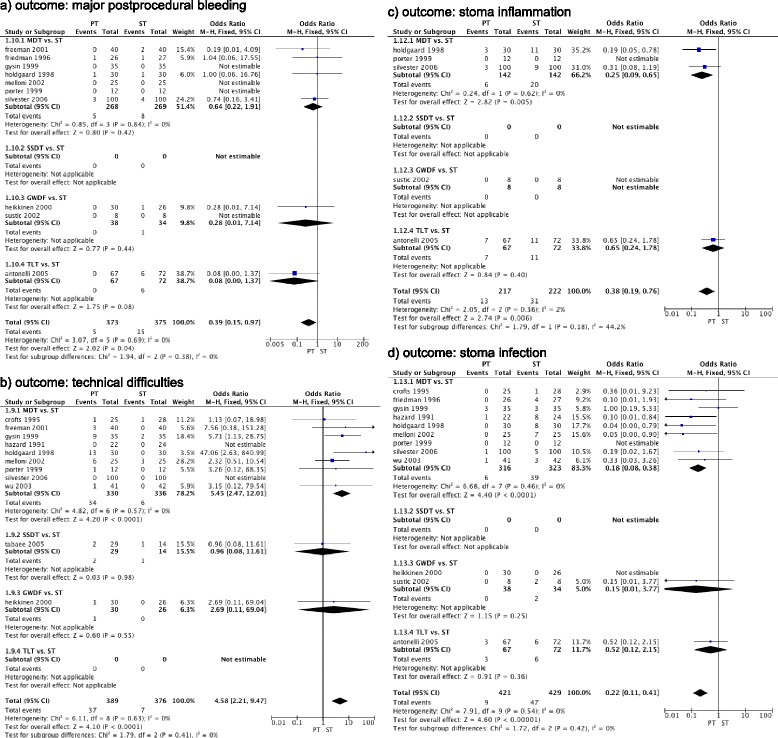
**Forest plot for comparison of percutaneous tracheostomy versus surgical tracheostomy. (a)** Outcome: major postprocedural bleeding. **(b)** Outcome: technical difficulties. **(c)** Outcome: stoma inflammation. **(d)** Outcome: stoma infection. CI, confidence interval; GWDF, guide wire dilatation forceps technique; MDT, multiple dilatator techniques; M-H, Mantel–Haenszel; PT, percutaneous tracheostomy; SSDT, single-step dilatator technique; ST, surgical tracheostomy; TLT, translaryngeal tracheostomy.

In the postprocedural period, PT techniques reduced odds for major bleeding (odds ratio, 0.39 (95% CI, 0.15 to 0.97 (*P* = 0.04)); *I*^2^ = 0% (*P* = 0.69)) (Figure 2a), stoma inflammation (odds ratio, 0.38 (95% CI, 0.19 to 0.76 (*P* = 0.006)); *I*^2^ = 2% (*P* = 0.36)) (Figure 2c), and infection (odds ratio, 0.22 (95% CI, 0.11 to 0.41 (*P* <0.00001)); *I*^2^ = 0% (*P* = 0.54)) (Figure 2d), but not for minor bleeding (odds ratio, 0.68 (95% CI, 0.38 to 1.24 (*P* = 0.21)); *I*^2^ = 0% (*P* = 0.52)) (Figure S1e in Additional file 3), and tracheal stenosis (odds ratio, 0.56 (95% CI, 0.18 to 1.76 (*P* = 0.32)); *I*^2^ = 0% (*P* = 0.71)) (Figure S1b in Additional file 3).

PT was associated with increased odds for procedural technical difficulties (odds ratio, 4.58 (95% CI, 2.21 to 9.47 (*P* <0.0001)); *I*^2^ = 0% (*P* = 0.63)) (Figure 2b). Odds for major intraprocedural bleeding (odds ratio, 2.12 (95% CI, 0.41 to 10.84 (*P* = 0.37)); *I*^2^ = 10% (*P* = 0.34)) (Figure S1a in Additional file 3), minor intraprocedural bleeding (odds ratio, 0.66 (95% CI, 0.25 to 1.77 (*P* = 0.41)); *I*^2^ = 52% (*P* = 0.03)) (Figure S1f in Additional file 3), false route (odds ratio, 2.17 (95% CI, 0.54 to 8.82 (*P* = 0.28)); *I*^2^ = 0% (*P* = 0.95)) (Figure S1g in Additional file 3), subcutaneous emphysema (odds ratio, 0.35 (95% CI, 0.05 to 2.26 (*P* = 0.27)); *I*^2^ = 0% (*P* = 1.00)) (Figure S1h in Additional file 3), pneumothorax (odds ratio, 1.07 (95% CI, 0.21 to 5.46 (*P* = 0.94)); *I*^2^ = 0% (*P* = 0.66)) (Figure S1i in Additional file 3), and desaturation (odds ratio, 1.09 (95% CI, 0.31 to 3.87 (*P* = 0.89)); *I*^2^ = 0% (*P* = 0.40)) (Figure S1j in Additional file 3) were not different between PT techniques and ST.

Odds for hospital survival (odds ratio, 1.04 (95% CI, 0.73 to 1.48 (*P* = 0.84)); *I*^2^ = 0% (*P* = 0.57)) (Figure S1c in Additional file 3) were not different between PT techniques and ST. Heterogeneity and the small sample size limited the interpretation of the procedure duration (mean reduction −13.06 minutes (95% CI, −19.37 to −6.76 (*P* <0.0001)); *I*^2^ = 97% (*P* <0.00001)) (Figure S1d in Additional file 3).

#### Multiple dilatator techniques versus surgical tracheostomy

Analysis of MDT versus ST included a total of 719 patients in 10 RCTs.

In the postprocedural period, MDT reduced odds for stoma inflammation (odds ratio, 0.25 (95% CI, 0.09 to 0.65 (*P* = 0.005)); *I*^2^ = 0% (*P* = 0.62)) (Figure 2c) and infection (odds ratio, 0.18 (95% CI, 0.08 to 0.38 (*P* <0.0001)); *I*^2^ = 0% (*P* = 0.46)) (Figure 2d), but not for major postprocedural bleeding (odds ratio, 0.64 (95% CI, 0.22 to 1.91 (*P* = 0.42)); *I*^2^ = 0% (*P* = 0.84)) (Figure 2a) or minor postprocedural bleeding (odds ratio, 0.58 (95% CI, 0.30 to 1.13 (*P* = 0.11)); *I*^2^ = 12% (*P* = 0.34)) (Figure S1e in Additional file 3) and tracheal stenosis (odds ratio, 0.57 (95% CI, 0.16 to 2.08 (*P* = 0.40)); *I*^2^ = 0% (*P* = 0.51)) (Figure S1b in Additional file 3).

PT was associated with increased odds for procedural technical difficulties (odds ratio, 5.45 (95% CI, 2.47 to 12.01 (*P* <0.0001)); *I*^2^ = 0% (*P* = 0.57)) (Figure 2b). Odds for major intraprocedural bleeding (odds ratio, 1.62 (95% CI, 0.18 to 14.50 (*P* = 0.67)); *I*^2^ = 34% (*P* = 0.22)) (Figure S1a in Additional file 3), minor intraprocedural bleeding (odds ratio, 0.68 (95% CI, 0.16 to 2.84 (*P* = 0.60)); *I*^2^ = 66% (*P* = 0.01)) (Figure S1f in Additional file 3), false route (odds ratio, 2.36 (95% CI, 0.50 to 11.07 (*P* = 0.28)); *I*^2^ = 0% (*P* = 0.82)) (Figure S1g in Additional file 3), subcutaneous emphysema (odds ratio, 0.35 (95% CI, 0.03 to 3.44 (*P* = 0.36)); *I*^2^ = 0% (*P* = 0.97)) (Figure S1h in Additional file 3), pneumothorax (odds ratio, 1.07 (95% CI, 0.21 to 5.46 (*P* = 0.94)); *I*^2^ = 0% (*P* = 0.66)) (Figure S1i in Additional file 3), and desaturation (odds ratio, 0.79 (95% CI, 0.30 to 21.00 (*P* = 0.89)); *I*^2^ = 65% (*P* = 0.09)) was not different between MDT and ST (Figure S1j in Additional file 3).

**Figure 3:**
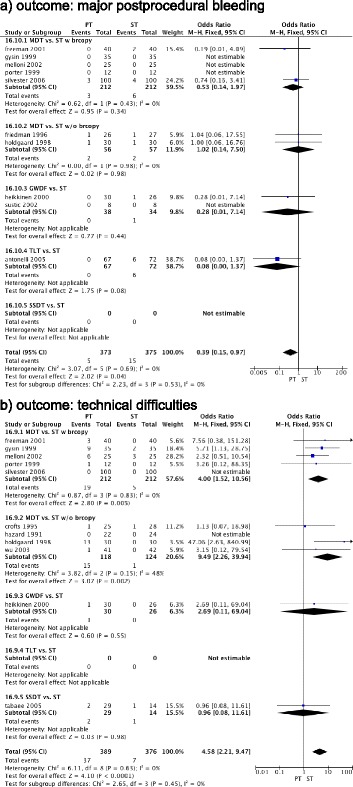
**Forest plot for comparison of percutaneous tracheostomy versus surgical tracheostomy with and without bronchoscopy. (a)** Outcome: major postprocedural bleeding. **(b)** Outcome: technical difficulties. CI, confidence interval; GWDF, guide wire dilatation forceps technique; MDT, multiple dilatator techniques; M-H, Mantel–Haenszel; PT, percutaneous tracheostomy; SSDT, single-step dilatator technique; ST, surgical tracheostomy; TLT, translaryngeal tracheostomy.

Odds for technical difficulties and major postprocedural bleeding were not different in a subgroup analysis comparing MDT versus ST with or without use of bronchoscopy (Figure 3a,b). Odds for hospital survival (odds ratio, 1.02 (95% CI, 0.67 to 1.54 (*P* = 0.93)); *I*^2^ = 0% (*P* = 0.43)) were not different between MDT and ST (Figure S1c in Additional file 3). Heterogeneity and the small sample size limited the interpretation of the length of the procedure (mean reduction of −15.69 minutes (95% CI, −22.96 to −8.43 (*P* <0.0001)); *I*^2^ = 97% (*P* <0.00001)) (Figure S1d in Additional file 3).

#### Pooled MDT + SSDT versus pooled GWDF + RDT + BDT

Analysis of pooled MDT + SSDT versus pooled GWDF + RDT + BDT included a total of 700 patients in eight RCTs.

**Figure 4:**
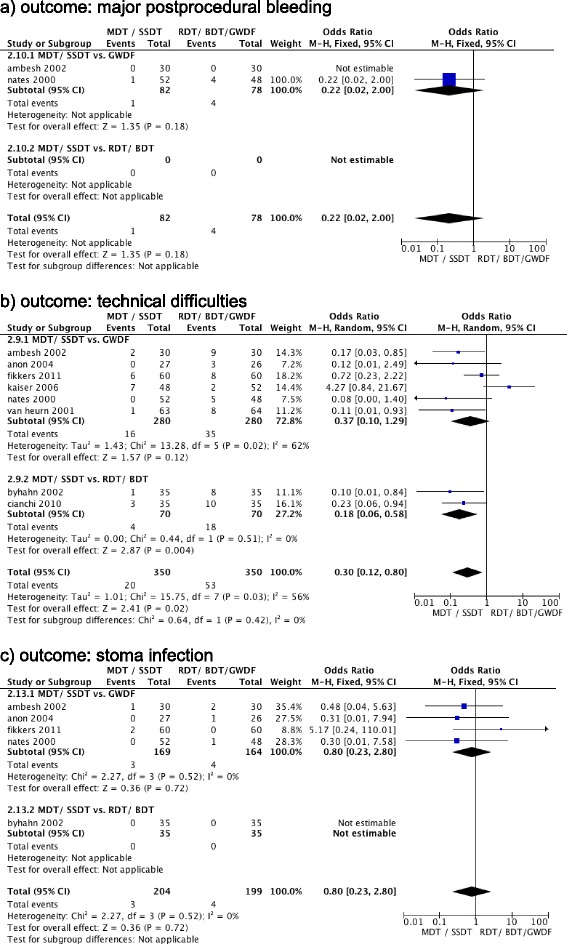
**Forest plot for comparison of multiple dilatator techniques**/**single-step dilatator technique versus rotational dilation tracheostomy**/**balloon dilation tracheostomy**/**guide wire dilatation forceps technique. (a)** Outcome: major postprocedural bleeding. **(b)** Outcome: technical difficulties. **(c)** Outcome: stoma infection. BDT, balloon dilation tracheostomy; CI, confidence interval; GWDF, guide wire dilatation forceps technique; MDT, multiple dilatator techniques; M-H, Mantel–Haenszel; PT, percutaneous tracheostomy; RDT, rotational dilation tracheostomy; SSDT, single-step dilatator technique; ST, surgical tracheostomy; TLT, translaryngeal tracheostomy.

Odds for stoma infection (odds ratio, 0.80 (95% CI, 0.23 to 2.80 (*P* = 0.72)); *I*^2^ = 0% (*P* = 0.52)) (Figure 4d), major postprocedural bleeding (odds ratio, 0.22 (95% CI, 0.20 to 2.00 (*P* = 0.18)); *I*^2^ = NA (P = NA)) (Figure 4a), minor postprocedural bleeding (odds ratio, 0.69 (95% CI, 0.08 to 5.98 (*P* = 0.74)); *I*^2^ = 86% (*P* = 0.007)) (Figure S2e in Additional file 3) and tracheal stenosis (odds ratio, 1.00 (95% CI, 0.14 to 7.22 (*P* = 1.00)); *I*^2^ = 0% (*P* = 0.34)) (Figure S2b in Additional file 3) were not different between pooled MDT + SSDT and pooled GWDF + RDT + BDT.

MDT + SSDT reduced the odds for intraprocedural technical difficulties (odds ratio, 0.30 (95% CI, 0.12 to 0.80 (*P* = 0.02)); *I*^2^ = 56% (*P* = 0.03)) (Figure 4b) and for major intraprocedural bleeding (odds ratio, 0.29 (95% CI, 0.10 to 0.85 (*P* = 0.02)); *I*^2^ = 0% (*P* = 0.72)) (Figure S2a in Additional file 3), but not for minor intraprocedural bleeding (odds ratio, 1.31 (95% CI, 0.50 to 3.47 (*P* = 0.58)); *I*^2^ = 53% (*P* = 0.07)) (Figure S2f in Additional file 3), false route (odds ratio, 0.24 (95% CI, 0.05 to 1.14 (*P* = 0.07)); *I*^2^ = 0% (*P* = 0.99)) (Figure S2g in Additional file 3), subcutaneous emphysema (odds ratio, 1.02 (95% CI, 0.12 to 8.92 (*P* = 0.99)); *I*^2^ = 30% (*P* = 0.24)) (Figure S2h in Additional file 3), pneumothorax (odds ratio, 3.21 (95% CI, 0.33 to 31.54 (*P* = 0.32)); *I*^2^ = 0% (*P* = 0.98)) (Figure S2i in Additional file 3), and desaturation (odds ratio, 1.02 (95% CI, 0.18 to 5.91 (*P* = 0.98)); *I*^2^ = 37% (*P* = 0.19)) (Figure S2j in Additional file 3) when compared with pooled GWDF + RDT + BDT.

Odds for hospital survival (odds ratio, 0.93 (95% CI, 0.44 to 2.00 (*P* = 0.86)); *I*^2^ = 33% (*P* = 0.22)) (Figure S2c in Additional file 3) and duration of the procedure (mean reduction of 0.64 minutes (95% CI, −0. 47 to 1.75 (*P* = 0.26)); *I*^2^ = 0% (*P* = 0.74)) (Figure S2d in Additional file 3) were not different between pooled MDT + SSDT and pooled GWDF + RDT + BDT.

#### Pooled MDT + SSDT versus GWDF

Analysis of pooled MDT + SSDT versus GWDF included a total of 560 patients in six RCTs. Only one RCT reported major or minor postprocedural bleeding [41] and one RCT reported hospital survival following tracheostomy [46].

Odds for stoma infection (odds ratio, 0.80 (95% CI, 0.23 to 2.80 (*P* = 0.72)); *I*^2^ = 0% (*P* = 0.52)) (Figure 4c) and tracheal stenosis (odds ratio, 0.33 (95% CI, 0.01 to 8.21 (*P* = 0.50)); *I*^2^ = NA) (Figure S2b in Additional file 3) were not different between pooled MDT + SSDT and GWDF.

MDT + SSDT reduced the odds for major intraprocedural bleeding (odds ratio, 0.29 (95% CI, 0.10 to 0.85 (*P* = 0.02)); *I*^2^ = 0% (*P* = 0.72)) (Figure S2a in Additional file 3), but not for minor intraprocedural bleeding (odds ratio, 1.26 (95% CI, 0.38 to 4.18 (*P* = 0.71)); *I*^2^ = 65% (*P* = 0.04)) (Figure S2f in Additional file 3), intraprocedural technical difficulties (odds ratio, 0.37 (95% CI, 0.10 to 1.29 (*P* = 0.12)); *I*^2^ = 62% (*P* = 0.02)) (Figure 4b), false route (odds ratio, 0.22 (95% CI, 0.04 to 1.31 (*P* = 0.10)); *I*^2^ = 0% (*P* = 0.96)) (Figure S2g in Additional file 3), subcutaneous emphysema (odds ratio, 0.62 (95% CI, 0.03 to 15.09 (*P* = 0.77)); *I*^2^ = 52% (*P* = 0.15)) (Figure S2h in Additional file 3), pneumothorax (odds ratio, 3.21 (95% CI, 0.33 to 31.54 (*P* = 0.32)); *I*^2^ = 0% (*P* = 0.98)) (Figure S2i in Additional file 3), and desaturation (odds ratio, 1.04 (95% CI, 0.09 to 12.53 (*P* = 0.98)); *I*^2^ = 58% (*P* = 0.09)) (Figure S2j in Additional file 3) when compared with GWDF.

Duration of the procedure (mean reduction of 0.64 minutes (95% CI, −0.47 to 1.75 (*P* = 0.26)); *I*^2^ = 0% (*P* = 0.74)) (Figure S2d in Additional file 3) was similar between pooled MDT + SSDT and pooled GWDF.

#### Publication bias

Macaskill’s modified test did not show publication bias for any considered outcome.

## Discussion

Available evidence from RCTs including adult critically ill patients tends to show that PT techniques are performed faster and reduce stoma inflammation and infection but are associated with increased technical difficulties when compared with ST. Among PT techniques, MDT + SSDT are associated with the lowest odds for intraprocedural technical difficulties and major bleeding, while GWDF accounts for increased odds for intraprocedural major bleeding.

### Percutaneous tracheostomy versus surgical tracheostomy

Previous meta-analyses comparing complication rates between PT techniques and ST reported conflicting results (Table 3). Dulguerov and colleagues analyzed 65 randomized and nonrandomized studies published between 1957 and 1996 and found higher incidence of perioperative complications, deaths, and cardiorespiratory arrests but less wound infection associated with PT when compared with historic control cohorts [48]. Cheng and Fee performed a systematic review of four studies including a total of 212 patients and found an increased rate of minor bleeding and stoma infection with ST [49]. Freeman and colleagues, pooling data from five RCTs including 236 patients, concluded that PT was associated with fewer complications, including bleeding and infections, and better survival [7]. Delaney and colleagues analyzed 17 RCTs including a total of 1,211 patients and found PT techniques to be equivalent to ST for bleeding and major periprocedural and long-term complications [6]. Higgins and Punthakee analyzed 15 RCTs and reported no clear difference but a trend towards fewer complications when using PT techniques [8]. The present meta-analysis was performed according to Cochrane Collaboration guidelines [21] including 14 RCTs with a total of 973 patients after strict quality assessment, and distinguished between complications occurring during and after the procedure.

**Table 3:** **Characteristics of precedent meta-analyses**

**Meta-analysis**	**Studies included**		**Sum of patients**	**Intervention**	**Endpoint**	**Results**	**Conclusions**	**Limitations**
Dulguerov and colleagues [48]	ST (1960 to 1984)	Total 4,185	• Serious complications: death, cardiopulmonary arrest, pneumothorax, pneumomediastinum, tracheoesophageal fistula, mediastinitis, sepsis, intratracheal postoperative hemorrhage, cannula obstruction and displacement, tracheal stenosis			• No OR, RR; or RD calculated	• Higher incidence of perioperative complications, perioperative death and serious cardiorespiratory events in the PT group	• Analyzes three historical cohorts
	ST (1985 to 1996)	Total 3,512	• Intermediate complications: intraoperative desaturation, lesions of the posterior tracheal wall, cannula misplacement, switch of a PT procedure to a surgical technique, aspiration, pneumonia, atelectasis, lesions of the tracheal cartilages				• Higher incidence of postoperative complications in the ST group	• Includes prospective and observational publications addressing perioperative and postoperative complications of tracheostomy
	PT	Total 1,817	• Mild complications: intraoperative hemorrhage, false passage, difficulty with tube placement, subcutaneous emphysema, postoperative wound hemorrhage, infections, delayed closure of tracheostomy tract, keloids, unaesthetic scarring					• Includes studies using different PT techniques to ST
								• Included studies comprise a variety of patient populations over a long period of time (1960 to 1996)
								• Does not follow the Cochrane Collaboration Guidelines
								• Does not distinguish between intraoperative and postoperative complications
Cheng and Fee [49]	Crofts and colleagues [31]		25/28	MDT vs. ST	*Intraoperative*	• No OR, RR or RD calculated	• Length of procedure is shorter in PT compared to ST	• Does not follow the Cochrane Collaboration Guidelines
	Friedman and colleagues [34]		26/27	MDT vs. ST	• Major bleeding		• Desaturation is less distinct in PT compared to ST	• Includes only four RCTs with small patient populations
	Hazard and colleagues [36]		22/24	MDT vs. ST	• Minor bleeding		• Lower incidence of minor intraoperative bleeding in PT	• No evaluation of long-term complications
	Holdgaard and colleagues [38]		30/30	MDT vs. ST	• Paratracheal insertion		• PT lower incidence of minor postoperative bleeding and infection	
			Total 103/109		• Difficult insertion			
					• Hypotension			
					• Desaturation			
					• Loss of airway			
					• Length of procedure			
					*Postoperative*:			
					• Major bleeding			
					• Minor bleeding			
					• Pneumothorax			
					• Subcutaneous emphysema			
					• Aspiration			
					• Atelectais			
					• Mortality			
Freeman and colleagues [7]	Hazard and colleagues [36]		22/24	MDT vs. ST	• Length of procedure	• Length of procedure: MD −9.8 (−7.83 to –11.85), *P* = NR, s	• PDT shorter length and greater ease of procedure	• Includes only five RCTs with small patient populations
	Crofts and colleagues [31]		25/28	MDT vs. ST	• Operative complications	• (All) operative complications: OR 0.73 (0.06 to 9.37), *P* = NR, ns	• PDT lower incidence of overall postoperative complications, intraprocedural and postprocedural bleeding and stoma infections	• No evaluation of long-term complications
	Friedman and colleagues [34]		26/27	MDT vs. ST	• Intraoperative bleeding	• Intraoperative bleeding: OR 0.15 (0.02 to 0.39), *P* = NR, s		• Evidence for publication bias
	Holdgaard and colleagues [38]		30/30	MDT vs. ST	• Postoperative complications	• (All) postoperative complications: OR 0.15 (0.07 to 0.29), *P* = NR, s		
	Porter and Ivatury [42]		12/12	MDT vs. ST	• Postoperative bleeding	• Postoperative bleeding: OR 0.39 (0.18 to 0.88), *P* = NR, s		
			Total 115/121		• Stoma infection	• Stoma infection: OR 0.02 (0.01 to 0.07), *P* = NR, s		
					• Mortality, not differentiated	• Mortality, not differentiated: OR 0.63 (0.18 to 2.20), *P* = NR, ns		
Delaney and colleagues [6]	Antonelli and colleagues [28]		67/72	TLT vs. ST	• Wound infection	• Wound infection OR 0.28 (0.16 to 0.49, *P* < 0.0005	• Compared with ST, PDT has a lower incidence of wound infections	• Does not distinguish between intraoperative and postoperative complications
	Crofts and colleagues [31]		25/28	MDT vs. ST	• Bleeding	• Bleeding OR 0.80 (0.45 to 1.41), *P* = 0.35	• Compared with ST, PT is not associated with a higher incidence of clinically significant bleeding, major periprocedural or long-term outcomes	• No evaluation of long-term complications
	Freeman and colleagues [33]		40/40	MDT vs. ST	• Mortality	• Mortality OR 0.79 (0.59 to 1.07), *P* = 0.13	• When comparing open ST performed in the OT versus PDT performed in the ICU, PDT has a lower incidence of relevant bleeding (*P* = 0.01) and mortality (*P* = 0.05)	
	Friedman and colleagues [34]		26/27	MDT vs. ST				
	Gysin and colleagues [35]		35/35	MDT vs. ST				
	Hazard and colleagues [36]		22/24	MDT vs. ST				
	Heikkinen and colleagues [37]		30/26	GWDF vs. ST				
	Holdgaard and colleagues [38]		30/30	MDT vs. ST				
	Ahn and colleagues [50]		NA/NA	MDT vs. ST				
	Massick and colleagues [51]		50/50	MDT vs. ST				
	Melloni and colleagues [40]		25/25	MDT vs. ST				
	Porter and Ivatury [42]		12/12	MDT vs. ST				
	Raine and colleagues [52]		50/50	GWDF vs. ST				
	Silvester and colleagues [43]		100/100	MDT vs. ST				
	Sustic and colleagues [44]		8/8	GWDF vs. ST				
	Tabaee and colleagues [45]		29/14	SSDT vs. ST				
	Wu and colleagues [47]		41/42	MDT vs. ST				
			Total 590/583	MDT vs. ST				
Higgins and Punthakee [8]	Antonelli and colleagues [28]		67/72	TLT vs. ST	• Minor hemorrhage	• Minor hemorrhage OR 1.09 (0.61 to 1.97), *P* = 0.77	• PDT higher incidence of false passage and accidental decannulation	• Does not distinguish between intraoperative and postoperative complications
	Crofts and colleagues [31]		25/28	MDT vs. ST	• Major hemorrhage	• Major hemorrhage OR 0.60 (0.28 to 1.26), *P* = 0.17	• PDT lower incidence of wound infection and unfavorable scarring	• Evidence for publication bias
	Freeman and colleagues [33]		40/40	MDT vs. ST	• False passage	• False passage OR 2.70 (0.89 to 8.22), *P* = 0.008	• PDT performed faster and with more cost-effectiveness	• Heterogeneous definition of study outcomes, in particular concerning bleeding and wound infection
	Friedman and colleagues [34]		26/27	MDT vs. ST	• Wound infection	• Wound infection OR 0.37 (0.22 to 0.62), *P* = 0.0002	• Overall complications did not differ between groups (*P* = 0.05)	
	Gysin and colleagues [35]		35/35	MDT vs. ST	• Unfavorable scar	• Unfavorable scar OR 0.44 (0.23 to 0.83), *P* = 0.01	• When comparing open TT performed in the OT vs. PDT performed in the ICU, PDT has a lower overall complication rate (*P* = 0.01)	
	Hazard and colleagues [36]		22/24	MDT vs. ST	• Decannulation/dislodgement	• Decannulation/obstruction OR 2.79 (1.29 to 6.03), *P* = 0.009		
	Heikkinen and colleagues [37]		30/26	GWDF vs. ST	• Subglottic stenosis	• Subglottic stenosis OR 0.59 (0.27 to 1.29), *P* = 0.19		
	Holdgaard and colleagues [38]		30/30	MDT vs. ST	• Mortality	• Mortality OR 0.70 (0.24 to 2.01), *P* = 0.50		
	Massick and colleagues [51]		50/50	MDT vs. ST				
	Melloni and colleagues [40]		25/25	MDT vs. ST				
	Porter and Ivatury [42]		12/12	MDT vs. ST				
	Raine and colleagues [52]		50/50	GWDF vs. ST				
	Sustic and colleagues [44]		8/8	GWDF vs. ST				
	Tabaee and colleagues [45]		29/14	SSDT vs. ST				
	Wu and colleagues [47]		41/42	MDT vs. ST				
			Total 490/483					
Cabrini and colleagues [15]	Anon and colleagues [27]		27/26	SSDT vs. GWDF	• Conversion to other method	• Conversion to other method	• SSDT lower incidence of mild complications than BDT and GWDF	• Only few studies, comparing different PT techniques; in particular those comparing TLT, BDT and RDT
	Ambesh and colleagues [26]		30/30	SSDT vs. GWDF	• Any mild complication	• TLT vs. GWDF RD = 23% (11 to 36%), *P* = 0.0002	• SSDT lower frequency of failure than RDT	• Does not distinguish between intraoperative and postoperative complications
	Birbicer and colleagues [53]		50/50	MDT vs. RDT	• Any severe complication	• SSDT vs. RDT RD = 17% (4 to 30%), *P* = 0.01	• GWDF lower incidence of severe complications and frequency of failure than TLT	
	Byhahn and colleagues [12]		25/25	MDT vs. SSDT		• Any mild complication	• No differences between MDT and SSDT	
	Byhahn and colleagues [29]		35/35	SSDT vs. RDT		• SSDT vs. BDT RD = 40% (22 to 58%), *P* < 0.0001	• MDT lower incidence of mild complications than GWDF, same incidence of severe complications and conversion rate	
	Cantais and colleagues [54]		47/53	TLT vs. GWDF		• SSDT vs. GWDF RD = 19% (5 to 33%), *P* = 0.008		
	Cianchi and colleagues [30]		35/35	SSDT vs. BDT		• Any severe complication		
	Johnson and colleagues [55]		25/25	MDT vs. SSDT		• TLT vs. GWDF RD = 30% (16 to 44%), *P* < 0.0001		
	Kaiser and colleagues [39]		48/42	MDT vs. GWDF				
	Nates and colleagues [41]		52/48	MDT vs. GWDF				
	Stocchetti and colleagues [56]		10/10	MDT vs. TLT				
	van Heurn and colleagues [46]		63/64	MDT vs. GWDF				
	Yurtseven and colleagues [57]		22/45	MDT vs. GWDF/RDT				
			Total 469/488					

BDT, balloon dilatation tracheotomy; GWDF, guide wire dilatation forceps; MD, weighted mean difference; MDT, multiple dilatation tracheotomy; NA, not available; NR, not reported; ns, not significant; OR, odds ratio; OT, operating theatre; RCT, randomized controlled trial; RD, risk difference; RDT, rotational dilatation tracheotomy; RR, relative risk; s, significant; SSDT, single-step dilatation tracheotomy; ST, surgical tracheotomy; PT, percutaneous tracheotomy; TLT, translaryngeal tracheostomy; TT, tracheostomy; PDT, percutaneous dilatation tracheotomy.

In agreement with previous meta-analysis, the present data did not demonstrate reduction of bleeding, false route, development of subcutaneous emphysema, occurrence of pneumothorax, and oxygen desaturation during PT when compared with ST [6–8,49]. Equivalent complications rates observed during PT and ST have been explained by differences in training status of the physicians, location where tracheostomy was performed, and different PT techniques. Higgins and Punthakee claimed that trainees are more likely to perform ST to learn the anatomy of the airway in the operative setting and then proceed to the PT where the airway is less well visualized [8]. In contrast with previous data [6], our systematic review does not support this belief because both ST and PT were performed by staff physicians and trainees. Two previous meta-analyses reported increased incidence of perioperative complications with ST when performed in the operating room but not at the bedside [6,8]. This was explained by difficulties and mishaps associated with transport to and from the operating room. In contrast, we found decreased risk of technical difficulties with ST regardless of whether ST was performed in the ICU or the operating room. Interestingly, we found no difference with regard to technical difficulties and odds for major postprocedural bleeding, regardless of whether bronchoscopy was used in PT techniques when compared with ST. Although transport of critically ill patients to and from the operating room may be associated with an increased risk of complications, no RCT reported increased transport related complication rates with ST. Because higher rates of technical difficulties were also observed with MDT + SSDT when compared with ST, it seems unlikely that the use of a specific PT technique could help to reduce procedural complications rates. This suggests that PT and ST techniques require adequate training and should always be performed by physicians able to promptly manage potential complications and mishaps.

In line with previous studies [6–8], we found PT techniques superior to reduce risk of postprocedural stoma inflammation and infection when compared with ST. The ability to have a tracheostomy that fits snugly in the stoma allowing compression of the surrounding tissues and the use of smaller incision and blunt dissection instead of cutting and transecting vessels may explain the less frequent and less severe inflammation and infection of the stoma following PT [6] and has been claimed to reduce postprocedural bleeding [48].

However, benefit of PT for decreasing postprocedural bleeding was discussed controversially. Other meta-analyses not distinguishing between intraoperative and postoperative bleeding did not observe reduction in bleeding following PT [8]. Although we observed a reduced risk of major postprocedural bleeding with PT techniques, from a statistical standpoint, some uncertainty may still exist regarding the benefit of PT techniques on postprocedural bleeding when compared with ST. When one RCT using TLT, which carries 39% of the weight in the pooled effect, was excluded from analysis [28], no advantage of PT could be demonstrated. MDT + SSDT were not associated with less major postprocedural bleeding when compared with ST. Based on the either limited number of available RCTs comparing TLT or GWDF with ST and the fact that no RCT compared RDT or BDT with ST, it cannot simply be concluded the PT *per se* is preventative for postprocedural bleeding.

Although tracheal stenosis, including subglottic stenosis, has been claimed to be a serious complication of PT that may require surgical repair [58], our data demonstrate a low and comparable incidence of tracheal stenosis with PT techniques and ST.

In 13 out of 14 RTCs, PT techniques were performed faster than ST. The pooled summary data indicating a faster procedure with PT than ST have to be viewed with caution because of the significant heterogeneity among studies (Table 2). We explored heterogeneity and found the limited number of included patients and the intra-individual variability of the procedure duration to be the major source for heterogeneity.

Our meta-analysis is the first to our knowledge demonstrating no difference in hospital mortality between PT and ST. This indicates that the observed differences in complication rates between PT and ST have no impact on survival rate.

### Differences among PT techniques

Six PT techniques using significantly different procedures have been introduced with the goal of simplifying the methods and to improve patient safety [9–14]. Recently, a meta-analysis including 13 RCTs comparing different PT techniques in 1,130 patients categorized mild and severe complications and could not demonstrate superiority of a single PT technique in terms of safety and success rate [15]. The present meta-analysis included eight RCTs with a total of 700 patients after strict quality assessment and distinguished between complications occurring during and after the procedure.

After a subgroup analysis comparing MDT with SSDT demonstrated no significant difference in complication rates and because of similarity of the procedures, we decided to compare pooled MDT + SSDT with other PT techniques. MDT + SSDT were associated with less risk of intraprocedural technical difficulties when compared with pooled GWDF + RDT + BDT but not when compared with GWDF alone. In contrast, GWDF accounted for an increased risk of major intraprocedural bleeding. These results may be explained by the GWDF technique using forceps for blunt dilatation of the pretracheal and intercartilaginous tissue after insertion of the guide wire into the trachea and skin incision. GWDF thus allows placement of the cannula in the trachea under direct vision, which may explain reduction of technical difficulties during tracheostomy tube insertion. However, the use of a forceps may be more traumatizing than the application of serial or single dilators.

Our meta-analysis demonstrates no difference in hospital mortality among PT techniques. This indicates that the observed differences in complication rates between PT techniques have no impact on survival rate.

Since only one RCT compared RDT or BDT with SSDT, no significant evidence is available to conclude on the safety and success rate of these recently introduced PT techniques.

### Limitations of the present analysis

There are a number of potential limitations to the present systematic review and meta-analysis that warrant discussion. When interpreting the present findings, it is important to consider that several groups of patients were excluded from the analyzed RCTs. Critically ill patients requiring emergency tracheostomy, with evidence or suspicion of difficult anatomy, prior airway problems, coagulopathies, and previous tracheostomy were generally excluded. Furthermore, length of stay and duration of mechanical ventilation prior to tracheostomy was not reported in most of the RCTs and was therefore excluded from analysis. In addition, some reported events and complications were rare. This may significantly limit the generalizability of the results of this meta-analysis to all adult critically ill patients. Furthermore, the effect of the experience of the physicians performing the tracheostomy could not be formally quantitatively assessed in the present analysis. Similarly, definitions of complications – for example, minor and major bleeding, stoma wound inflammation and/or infection, and technical difficulties – were not standardized and varied from center to center. Finally, formal statistical tests did not support the presence of publication bias for any considered outcome, which could have had an impact on the pooled effect estimates.

## Conclusion

On the basis of available evidence from RCTs in critically ill adult patients, PT techniques can be performed faster and reduce stoma inflammation and infection but are associated with increased technical difficulties when compared with ST. Combined MDT + SSDT was associated with the lowest risk of intraprocedural technical difficulties and bleeding, and therefore seem to be the preferable PT technique in critically ill adult patients.

## Key messages

- PT can be performed faster and reduces the risk for stoma inflammation and infection when compared with ST.
- PT is associated with increased technical difficulties when compared with ST.
- Among PT techniques, MDT and SSDT were associated with the lowest risk of intraprocedural technical difficulties and major intraprocedural bleeding.
- Risk for tracheal stenosis and odds for hospital survival were not different between PT techniques and ST.

## Additional files

Additional file 1: Table S1. Presenting a description of different percutaneous tracheostomy techniques. Additional file 2: Table S2. Presenting characteristics of the study population and exclusion criteria in the included trials. Additional file 3: Figures S1 and S2. Showing the effect of different tracheostomy techniques on complications and outcome – intraprocedural and postprocedural complications.

## Abbreviations

BDT: balloon dilation tracheostomy
CI: confidence interval
GWDF: guide wire dilatation forceps technique
MDT: multiple dilatator techniques
PT: percutaneous tracheostomy
RCT: randomized controlled trial
RDT: rotational dilation tracheostomy
SSDT: single-step dilatator technique
ST: surgical tracheostomy
TLT: translaryngeal tracheostomy

## References

[1] Brambrink A (2004). Percutaneous dilatation tracheostomy: which technique is the best for the critically ill patient, and how can we gather further scientific evidence?. Crit Care.

[2] Carrer S, Basilico S, Rossi S, Bosu A, Bernorio S, Vaghi GM (2009). Outcomes of percutaneous tracheostomy. Minerva Anestesiol.

[3] Guarino A (2009). Percutaneous tracheostomy: patient outcomes. It is always time to improve our care. Minerva Anestesiol.

[4] Arabi YM, Alhashemi JA, Tamim HM, Esteban A, Haddad SH, Dawood A, Shirawi N, Alshimemeri AA (2009). The impact of time to tracheostomy on mechanical ventilation duration, length of stay, and mortality in intensive care unit patients. J Crit Care.

[5] Veenith T, Ganeshamoorthy S, Standley T, Carter J, Young P (2008). Intensive care unit tracheostomy: a snapshot of UK practice. Int Arch Med.

[6] Delaney A, Bagshaw SM, Nalos M (2006). Percutaneous dilatational tracheostomy versus surgical tracheostomy in critically ill patients: a systematic review and meta-analysis. Crit Care.

[7] Freeman BD, Isabella K, Lin N, Buchman TG (2000). A meta-analysis of prospective trials comparing percutaneous and surgical tracheostomy in critically ill patients. Chest.

[8] Higgins KM, Punthakee X (2007). Meta-analysis comparison of open versus percutaneous tracheostomy. Laryngoscope.

[9] Ciaglia P, Firsching R, Syniec C (2009). Elective percutaneous dilatational tracheostomy. A new simple bedside procedure; preliminary report. 1985. Chest.

[10] Griggs WM, Worthley LI, Gilligan JE, Thomas PD, Myburg JA (1990). A simple percutaneous tracheostomy technique. Surg Gynecol Obstet.

[11] Fantoni A, Ripamonti D (1997). A non-derivative, non-surgical tracheostomy: the translaryngeal method. Intensive Care Med.

[12] Byhahn C, Wilke HJ, Halbig S, Lischke V, Westphal K (2000). Percutaneous tracheostomy: ciaglia blue rhino versus the basic ciaglia technique of percutaneous dilational tracheostomy. Anesth Analg.

[13] Frova G, Quintel M (2002). A new simple method for percutaneous tracheostomy: controlled rotating dilation. A preliminary report. Intensive Care Med.

[14] Zgoda MA, Berger R (2005). Balloon-facilitated percutaneous dilational tracheostomy tube placement: preliminary report of a novel technique. Chest.

[15] Cabrini L, Monti G, Landoni G, Biondi-Zoccai G, Boroli F, Mamo D, Plumari VP, Colombo S, Zangrillo A: **Percutaneous tracheostomy, a systematic review.** *Acta Anaesthesiol Scand* 2012, **56:**270**–**281. 10.1111/j.1399-6576.2011.02592.x 22188176

[16] **ClinicalTrials.gov.** [http://www.clinicaltrials.gov]

[17] **Clinical Study Results.** [www.clinicaltrialresults.org]

[18] Peters JL, Sutton AJ, Jones DR, Abrams KR, Rushton L (2006). Comparison of two methods to detect publication bias in meta-analysis. JAMA.

[19] Denzin NRC (1995). Narrative analysis. J Commun.

[20] Dixon-Woods M, Shaw RL, Agarwal S, Smith JA (2004). The problem of appraising qualitative research. Qual Saf Health Care.

[21] Higgins JPT GSe: *Cochrane Handbook for Systematic Reviews of Interventions Version 5.1.0* [updated March 2011]. Oxford, UK: The Cochrane Collaboration, 2011.

[22] Higgins JP, Thompson SG (2002). Quantifying heterogeneity in a meta-analysis. Stat Med.

[23] Higgins JP, Thompson SG, Deeks JJ, Altman DG (2003). Measuring inconsistency in meta-analyses. BMJ.

[24] Deeks JJ (2001). Systematic reviews in health care: systematic reviews of evaluations of diagnostic and screening tests. BMJ.

[25] Fleiss JLCJ (1973). The equivalence of weighted kappa and the intraclass correlation coefficient as measures of reliability. Educ Psychol Meas.

[26] Ambesh SP, Pandey CK, Srivastava S, Agarwal A, Singh DK: **Percutaneous tracheostomy with single dilatation technique: a prospective, randomized comparison of Ciaglia Blue Rhino versus Griggs' guidewire dilating forceps.** *Anesth Analg* 2002, **95:**1739–1745; table of contents. 10.1097/00000539-200212000-00050 12456450

[27] Anon JM, Escuela MP, Gomez V, Moreno A, Lopez J, Diaz R, Montejo JC, Sirgo G, Hernandez G, Martinez R (2004). Percutaneous tracheostomy: Ciaglia Blue Rhino versus Griggs' guide wire dilating forceps. A prospective randomized trial. Acta Anaesthesiol Scand.

[28] Antonelli M, Michetti V, Di Palma A, Conti G, Pennisi MA, Arcangeli A, Montini L, Bocci MG, Bello G, Almadori G, Paludetti G, Proietti R (2005). Percutaneous translaryngeal versus surgical tracheostomy: a randomized trial with 1-yr double-blind follow-up. Crit Care Med.

[29] Byhahn C, Westphal K, Meininger D, Gurke B, Kessler P, Lischke V (2002). Single-dilator percutaneous tracheostomy: a comparison of PercuTwist and Ciaglia Blue Rhino techniques. Intensive Care Med.

[30] Cianchi G, Zagli G, Bonizzoli M, Batacchi S, Cammelli R, Biondi S, Spina R, Peris A: **Comparison between single-step and balloon dilatational tracheostomy in intensive care unit: a single-centre, randomized controlled study.** *Br J Anaesth* 2010, **104:**728**–**732. 10.1093/bja/aeq087 20413380

[31] Crofts SL, Alzeer A, McGuire GP, Wong DT, Charles D (1995). A comparison of percutaneous and operative tracheostomies in intensive care patients. Can J Anaesth.

[32] Fikkers BG, Staatsen M, van den Hoogen FJ, van der Hoeven JG: **Early and late outcome after single step dilatational tracheostomy versus the guide wire dilating forceps technique: a prospective randomized clinical trial.** *Intensive Care Med* 2011, **37:**1103**–**1109. 10.1007/s00134-011-2222-4 PMC3127000 21484081

[33] Freeman BD, Isabella K, Cobb JP, Boyle WA, Schmieg RE, Kolleff MH, Lin N, Saak T, Thompson EC, Buchman TG (2001). A prospective, randomized study comparing percutaneous with surgical tracheostomy in critically ill patients. Crit Care Med.

[34] Friedman Y, Fildes J, Mizock B, Samuel J, Patel S, Appavu S, Roberts R (1996). Comparison of percutaneous and surgical tracheostomies. Chest.

[35] Gysin C, Dulguerov P, Guyot JP, Perneger TV, Abajo B, Chevrolet JC (1999). Percutaneous versus surgical tracheostomy: a double-blind randomized trial. Ann Surg.

[36] Hazard P, Jones C, Benitone J (1991). Comparative clinical trial of standard operative tracheostomy with percutaneous tracheostomy. Crit Care Med.

[37] Heikkinen M, Aarnio P, Hannukainen J (2000). Percutaneous dilational tracheostomy or conventional surgical tracheostomy?. Crit Care Med.

[38] Holdgaard HO, Pedersen J, Jensen RH, Outzen KE, Midtgaard T, Johansen LV, Moller J, Paaske PB (1998). Percutaneous dilatational tracheostomy versus conventional surgical tracheostomy. A clinical randomised study. Acta Anaesthesiol Scand.

[39] Kaiser E, Cantais E, Goutorbe P, Salinier L, Palmier B (2006). Prospective randomized comparison of progressive dilational vs forceps dilational percutaneous tracheostomy. Anaesth Intensive Care.

[40] Melloni G, Muttini S, Gallioli G, Carretta A, Cozzi S, Gemma M, Zannini P: **Surgical tracheostomy versus percutaneous dilatational tracheostomy. A prospective-randomized study with long-term follow-up.** *J Cardiovasc Surg (Torino)* 2002, **43:**113–121. 11803342

[41] Nates JL, Cooper DJ, Myles PS, Scheinkestel CD, Tuxen DV (2000). Percutaneous tracheostomy in critically ill patients: a prospective, randomized comparison of two techniques. Crit Care Med.

[42] Porter JM, Ivatury RR (1999). Preferred route of tracheostomy – percutaneous versus open at the bedside: a randomized, prospective study in the surgical intensive care unit. Am Surg.

[43] Silvester W, Goldsmith D, Uchino S, Bellomo R, Knight S, Seevanayagam S, Brazzale D, McMahon M, Buckmaster J, Hart GK, Opdam H, Pierce RJ, Gutteridge GA (2006). Percutaneous versus surgical tracheostomy: a randomized controlled study with long-term follow-up. Crit Care Med.

[44] Sustic A, Krstulovic B, Eskinja N, Zelic M, Ledic D, Turina D: **Surgical tracheostomy versus percutaneous dilational tracheostomy in patients with anterior cervical spine fixation: preliminary report.** *Spine (Phila Pa 1976)* 2002, **27:**1942–1945; discussion 1945. 10.1097/00007632-200209010-00026 12221364

[45] Tabaee A, Geng E, Lin J, Kakoullis S, McDonald B, Rodriguez H, Chong D (2005). Impact of neck length on the safety of percutaneous and surgical tracheotomy: a prospective, randomized study. Laryngoscope.

[46] Van Heurn LW, Mastboom WB, Scheeren CI, Brink PR, Ramsay G (2001). Comparative clinical trial of progressive dilatational and forceps dilatational tracheostomy. Intensive Care Med.

[47] Wu JJ, Huang MS, Tang GJ, Shih SC, Yang CC, Kao WF, Huang MH, Lee CH (2003). Percutaneous dilatational tracheostomy versus open tracheostomy – a prospective, randomized, controlled trial. J Chin Med Assoc.

[48] Dulguerov P, Gysin C, Perneger TV, Chevrolet JC (1999). Percutaneous or surgical tracheostomy: a meta-analysis. Crit Care Med.

[49] Cheng E, Fee WE (2000). Dilatational versus standard tracheostomy: a meta-analysis. Ann Otol Rhinol Laryngol.

[50] Ahn JJ, Koh YS, Chin JY, Lee KM, Park W, Hong SB, Shim TS, Lee SD, Kim WS, Kim DS, Kim WD, Lim CM (1998). Comparison of clinical efficacy between percutaneous dilatational tracheostomy and surgical tracheostomy. Tuberculosis Respiratory Dis.

[51] Massick DD, Yao S, Powell DM, Griesen D, Hobgood T, Allen JN, Schuller DE (2001). Bedside tracheostomy in the intensive care unit: a prospective randomized trial comparing open surgical tracheostomy with endoscopically guided percutaneous dilational tracheotomy. Laryngoscope.

[52] Raine R MW, Ruttmann TG, Bloch MB, Thorp M, Gough AM: **Late outcome after guide-wire forceps percutaneous tracheostomy – a prospective, randomised comparison with open surgical tracheostomy.** *Br J Anaesth* 1999, **82:**68.

[53] Birbicer H, Doruk N, Yapici D, Atici S, Altunkan AA, Epozdemir S, Oral U (2008). Percutaneous tracheostomy: a comparison of PercuTwist and multi-dilatators techniques. Ann Card Anaesth.

[54] Cantais E, Kaiser E, Le-Goff Y, Palmier B (2002). Percutaneous tracheostomy: prospective comparison of the translaryngeal technique versus the forceps-dilational technique in 100 critically ill adults. Crit Care Med.

[55] Johnson JL, Cheatham ML, Sagraves SG, Block EF, Nelson LD (2001). Percutaneous dilational tracheostomy: a comparison of single- versus multiple-dilator techniques. Crit Care Med.

[56] Stocchetti N, Parma A, Songa V, Colombo A, Lamperti M, Tognini L (2000). Early translaryngeal tracheostomy in patients with severe brain damage. Intensive Care Med.

[57] Yurtseven N, Aydemir B, Karaca P, Aksoy T, Komurcu G, Kurt M, Ozkul V, Canik S (2007). PercuTwist: a new alternative to Griggs and Ciaglia's techniques. Eur J Anaesthesiol.

[58] Raghuraman G, Rajan S, Marzouk JK, Mullhi D, Smith FG (2005). Is tracheal stenosis caused by percutaneous tracheostomy different from that by surgical tracheostomy?. Chest.

