# Supplementary material for: Percutaneous and surgical tracheostomy in critically ill adult patients: a meta-analysis
Source: Crit Care. 2014 Dec 19;18(6):544. doi: 10.1186/s13054-014-0544-7 (PMC4293819; doi:10.1186/s13054-014-0544-7)
Supplement: Additional file 1: Table S1. — Presenting a description of different percutaneous tracheostomy techniques. [file 13054_2014_544_MOESM1_ESM.pdf]

Table S1. **Description of percutaneous tracheostomy techniques**

Abbreviation	Technique	Synonym	Introduced/ year	Description
				All percutaneous tracheostomy techniques consist of inserting a tracheostomy tube using a Seldinger guidewire technique. Distinct methods differ in the process of dilatation and insertion of the tracheostomy tube.
**Antegrade techniques**				After insertion of the guidewire, the *antegrade* techniques dilate the tissue from the skin towards the trachea using one or more dilator-introducers, a forceps, or a balloon-technique
**MDT**	Multiple dilator tracheostomy	Ciaglia	1985	The primary “Ciaglia technique uses a multistep dilatation procedure with tapered dilators.<br>Following the guidewire, dilators of increasing size progressively enlarge the opening in the tracheal wall.<br>In a last step, a tracheostomy tube snugly fitted over the last dilator is passed into the trachea between the cricoid cartilage and the first tracheal ring.
**GWDF**	Guide wire dilating forceps	Griggs	1990	The “Griggs technique” uses a modified (curved) Howard Kelly forceps to dilate the soft tissue and then the trachea, or both at once. After passing the guidewire through the hole in the tip of the forceps and advancing it until it is inside the tracheal lumen, the forceps is opened and withdrawn with both hands. After such dilation of the anterior tracheal wall, the forceps is removed and the tracheostomy tube is passed over the guidewire into the trachea.
**SSDT**	Single-step dilator tracheostomy	Ciaglia blue rhino™	1999	The “Ciaglia blue rhino™ technique” uses a single-step dilatation with a curved dilator and a (tracheostomy-tube-) loading dilator.<br>Following the guidewire, a single tapered, horn shaped dilator enlarges the opening in the tracheal wall. After complete dilatation,the dilator is removed and the tracheostomy tube is passed over the guidewire into the trachea.
**RDT**	Rotational dilatation tracheostomy	Percutwist™	2002	The “Percutwist™ technique” uses a specially designed screw-type dilator with a thread to facilitate dilation of soft tissue and tracheal wall (“controlled rotating dilation”). After complete dilatation, the dilator is twisted back, removed and the tracheostomy tube is advanced over the guidewire into the trachea.
**BDT**	Balloon dilatation tracheostomy	Ciaglia blue dolphin™	2005	The “Ciaglia blue dolphin™ technique” uses a balloon-tipped dilatation-catheter, an inflation device and a (tracheostomy-tube-) loading dilator.<br>After complete pre-dilation and removal of the first, short dilator, the balloon-catheter/ loading dilator assembly is introduced into the trachea.<br>Using the inflation pump, the balloon is filled with normal saline until a maximum pressure of 11 atm for 15 s. After deflation of the balloon, the whole balloon-catheter/ loading dilator/ tracheostomy tube assembly is advanced as a unit into the trachea until the tracheostomy tube is in place and the rest of the assembly can be removed.
**Retrograde technique**				After insertion of the guidewire, the *retrograde* technique dilates the tissue from the trachea towards the skin using a specific “cone-cannula”.
**TLT**	Translaryngeal tracheostomy	Fantoni	1997	This method uses a special flexible plastic cone with a pointed metal tip joined to an armoured tracheal cannula (“cone-cannula”), acting as both dilator and tracheostomy tube. The initial puncture of the trachea is carried out with the needle directed cranially under bronchoscopic guidance. The cone-cannula is anchored to the guidewire and pulled through the orotracheal route. When the metallic cone passed through the skin, it is cut off and the cannula is then rotated downwards using a plastic obturator. This technique always requires removing the endotracheal tube in place and the use a specific cuffed endotracheal catheter to oxygenate and ventilate the patient to perform the maneuver.

MDT = multiple dilatation tracheotomy (Ciaglia); SSDT = single step dilatation tracheotomy (blue rhino); TLT = translaryngeal tracheotomy (Fantoni); RDT = rotational dilatation tracheotomy (percutwist); BDT = balloon dilatation tracheotomy (blue dolphin); GWDF = guide wire dilatation forceps (Griggs); atm. = atmosphere (760 Torr)
