# Supplementary material for: Percutaneous and surgical tracheostomy in critically ill adult patients: a meta-analysis
Source: Crit Care. 2014 Dec 19;18(6):544. doi: 10.1186/s13054-014-0544-7 (PMC4293819; doi:10.1186/s13054-014-0544-7)
Supplement: Additional file 2: Table S2. — Presenting characteristics of the study population and exclusion criteria in the included trials. [file 13054_2014_544_MOESM2_ESM.pdf]

Table S2. **Characteristics of study population, exclusion criteria and outcomes**

study	patient population	inter-vention	com-parison	exclusion criteria	severity of illness	outcome/ complications	performing medical specialty	location of procedure	use of bronchos-copy	length of procedure (min)
Wu 2003 [45]	mixed general ICU patients	MDT	ST	previous neck surgery, difficult anatomy	NR	intraoperative bleeding, tube dislocation, technical difficulties, difficult dilatation, postoperative bleeding, stoma infection, length of procedure, tracheal stenosis, tracheomalacia, hospital mortality	MDT: surgeon ST: surgeon	MDT: ICU ST: OR	no	22 (12,1) vs. 41,5 (5,9)
Freeman 2001 [31]	mixed medical, neurological, trauma and surgical patients	MDT	ST	difficult anatomy, refractory coagulopathy, difficult airway for translaryngeal intubation	controlled: APACHE II	intraoperative bleeding, tube dislocation, technical difficulties, difficult dilatation, postoperative bleeding, length of procedure, LOS ICU, LOS hospital, ICU mortality, costs of procedure	NR	MDT: ICU ST: OR	yes, in MDT	20,10 (2) vs. 41,7 (3,9)
Hazard 1991 [34]	mixed medical, neurological, trauma and surgical patients	MDT	ST	age < 15 yrs., potential extubation within 4 days, uncorrectable hemostatic defects, anatomic distortion of the trachea (by tumor, thyromegaly, scarring by previous TT), infection of the soft tissues of the neck	controlled: SAPS II	intraoperative bleeding, pneumothorax, technical difficulties, difficult insertion, postoperative bleeding, stoma infection, tracheal stenosis, length of procedure, hospital mortality	MDT: nr ST: surgeon	MDT: ICU ST: ICU or OR	no	4,3 (2,2) vs. 13,5 (7,3)
Crofts 1995 [29]	mixed medical, surgical and neurosurgical patients	MDT	ST	age < 16 yrs., enlarged thyroid gland, previous TT, cervical spine fracture, coagulopathy	controlled: APACHE II	intraoperative bleeding, sc emphysema, pneumothorax, technical difficulties, difficult insertion, stoma infection, ICU mortality	MDT: ENT ST: surgeon	MDT: ICU ST: OR	no	NR
Friedman 1996 [32]	mixed medical, surgical and trauma patients	MDT	ST	clinical instability, PEEP >15 cmH_2O, uncorrectable coagulopathy, previous TT or neck surgery, thyromegaly, anatomic distortion of trachea, skin/ soft tissue infection at the TT site	controlled: APACHE II	intraoperative bleeding, desaturation, sc emphysema, false route, tube disocation, postoperative bleeding, tube dislocation, stoma infection, length of procedure, hospital mortality	MDT: intensivist ST: surgeon	MDT: ICU ST: OR	no	8,2 (4,9) vs. 33,9 (14)
Porter 1999 [40]	mixed medical and surgical patients	MDT	ST	emergency TT, coagulopathy	NR	intraoperative bleeding, desaturation, pneumothorax, tube disocation, technical difficulties, difficult insertion, postoperative bleeding, accidental decannulation, stoma inflammation, stoma infection, length of procedure	MDT: surgeon ST: surgeon	MDT: ICU ST: ICU or OR	yes, in MDT	15,4 (3,8) vs. 25,2 (9,5)
Gysin 1999 [33]	NR	MDT	ST	previous TT, tracheal pathology	NR	intraoperative bleeding, desaturation, sc emphysema, pneumothorax, false route, technical difficulties, difficult dilatation, difficult insertion, postoperative bleeding, accidental decannulation, stoma infection, tracheal stenosis, pneumonia, length of procedure	MDT: ENT ST: ENT	MDT: ICU ST: ICU or OR	yes, in MDT	18,2 (11,2) vs. 15,8 (5,5)
Silvester 2006 [41]	mixed medical, neurological, trauma and surgical patients	MDT	ST	coagulopathy, anatomical abnormality of the anterior neck (trachea, thyroid, vessels), previous TT, cervical spine injury	controlled: APACHE II	intraoperative bleeding, desaturation, hypercarbia, pneumothorax, false route, tube disocation, technical difficulties, postoperative bleeding, stoma inflammation, stoma infection, tracheal stenosis, length of procedure, ICU mortality, hospital mortality	MDT: intensivist ST: surgeon	MDT: ICU ST: ICU	yes, in MDT	20 (nr) vs. 17 (nr)
Holdgaard 1998 [36]	mixed medical, neurological and surgical patients	MDT	ST	age < 18 yrs., previous TT, pathology of the neck, neck deformities, unidentifiable anatomy of the neck, pts. with radiotherapy	NR	intraoperative bleeding, desaturation, sc emphysema, pneumothorax, false route, technical difficulties, difficult insertion, postoperative bleeding, stoma inflammation, stoma infection, length of procedure, LOS hospital	MDT: nr ST: surgeon	MDT: OR ST: OR	no	median 11,5 vs. median 15,5

Melloni 2002 [38]	mixed medical, trauma and neurological patients	MDT	ST	age <15 yrs., intubation >10d, thyromegaly, history of laryngeal and tracheal disease, previous TT or neck, laryngeal and tracheal surgery	controlled: SAPS	intraoperative bleeding, false route, , technical difficulties, difficult insertion, postoperative bleeding, accidental decannulation, stoma infection, tracheal stenosis, length of procedure, ICU mortality, hospital mortality	MDT: intensivist ST: surgeon	MDT: ICU ST: ICU or OR	yes, in MDT	14 (6) vs. 41 (14)
Sustic 2002 [42]	pts. after spinal cord fusion for spinal cord trauma	GWDF	ST	infection of the surgical scar, refractory coagulopathy	NR	intraoperative bleeding, desaturation, hypercarbia, postoperative infection, postoperative bleeding, stoma inflammation, stoma infection, length of procedure, LOS ICU, ICU mortality	GWDF: intensivist ST: surgeon	GWDF: ICU ST: OR	no	8 (6) vs. 21 (7)
Antonelli 2005 [26]	mixed medical, trauma and surgical patients	TLT	ST	emergency TT, age <18 yrs, severe coagulopathy, surgical wounds near the TT site, previous TT, difficult anatomy, inability to extend the neck, significant thyroid gland enlargement, palpable neck vessels	controlled: SAPS II	intraoperative bleeding, desaturation, sc emphysema, tube dislocation, postoperative bleeding, stoma inflammation, stoma infection, tracheomalacia, tracheal stenosis, pneumonia, LOS ICU, length of prcedure, ICU mortality, hospital mortality	TLT: intensivist ST: ENT	TLT: ICU ST: OR	no	17 (10) vs. 22 (6)
Tabaee 2005 [43]	medical and neurological patients	SSDT	ST	NR	NR	intraoperative bleeding, desaturation, false route, tube dislocation, technical difficulties, difficult dilatation, difficult insertion, postoperative bleeding, length of procedure	MDT: intensivist/ ENT ST: ENT	MDT: ICU ST: ICU	yes, in MDT	8 (nr) vs. 23,6 (nr)
Heikkinen 2000 [35]	surgical patients	GWDF	ST	previous TT or neck surgery, thyromegaly, infection at the TT site, extreme obesity	NR	intraoperative bleeding, technical difficulties, difficult insertion, postoperative bleeding, stoma infection, length of procedure	GWDF:surgeon ST: surgeon	GWDF:ICU ST: ICU	no	11,13 (6,4) vs. 14,4 (6)
Byhahn 2002 [27]	mixed medical, neurological and surgical patients	SSDT	RDT	infection of the TT site, difficult endotracheal intubation, difficult anatomy, instable cervical spine, age <18 yrs.	NR	intraoperative bleeding, desaturation, sc emphysema, false route, tube dislocation, technical difficulties, difficult dilatation, difficult insertion, postoperative bleeding, accidental decannulation, staom inflammation, stoma infection, length of procedure	SSDT: intensivist RDT: intensivist	SSDT:ICU RDT: ICU	yes, in SSDT and RDT	NR
Cianchi 2010 [28]	mixed medical and trauma patients	SSDT	BDT	infection, injury or malignancies of the anterior neck, severe headinjury, with CPP <60 mmHg despite therapy, thyromegaly, coagulation disorders	NR	intraoperative bleeding, desaturation, pneumothorax, false route, difficult insertion, postoperative bleeding, tracheal stenosis, LOS ICU, LOS hospital, length of procedure, ICU mortality, hospital mortality	SSDT: intensivist BDT: intensivist	SSDT: ICU BDT: ICU	yes, in SSDT and BDT	median 1,5 vs. median 4
van Heurn 2001 [44]	NR	MDT	GWDF	PEEP >10cmH_2O, FiO_2 >0.8, uncorrectable coagulopathy, infection of the soft tissues of the neck, fracture of the cervical spine	NR	intraoperative bleeding, false route, technical difficulties, difficult insertion, LOS ICU, hospital mortality	MDT: surgeon GWDF: surgeon	MDT: ICU GWDF: ICU	no	7 (3,5) vs. 6,4 (4,9)
Ambesh 2002 [24]	NR	SSDT	GWDF	NR	controlled: APACHE II	intraoperative bleeding, desaturation, hypercarbia, sc emphysema, pneumothorax, technical difficulties, difficult insertion, postoperative bleeding, tube dislocation, stoma infection, length of procedure	SSDT: intensivist GWDF: intensivist	SSDT: ICU GWDF: ICU	yes	7,5 (2,5) vs. 6,5 (4,5)
Nates 2000 [39]	mixed trauma and general ICU patients	MDT	GWDF	infection of the anterior neck, uncontrolled coagulopathy, age <15 yrs., anatomical abnormalities (enlarged thyroid gland or vascular abnromalities), airway emergencies, previous neck surgery or trauma, pregnancy, burns, poststernotomy	controlled: APACHE II	intraoperative bleeding, pneumothorax, false route, technical difficulties, postoperative bleeding, stoma infection, length of procedure, ICU mortality	MDT: intensivist GWDF: intensivist	MDT: ICU GWDF: ICU	no	9,3 (7) vs. 10 (12)
Anon 2004 [25]	mixed medical, neurological, trauma and surgical patients	SSDT	GWDF	PEEP >10cmH_2O, FiO_2 >0.8, uncorrectable coagulopathy, infection of the soft tissues of the neck, fracture of the cervical spine, anatomical distortion of the trachea, previous neck surgery, goiter, infection of the TT site	controlled: APACHE II	intraoperative bleeding, desaturation, sc emphysema, pneumothorax, false route, technical difficulties, difficult insertion, stoma infection, tracheal stenosis, length of procedure, ICU mortality	SSDT: intensivist GWDF: intensivist	SSDT: ICU GWDF: ICU	no	median 9 vs. median 7

Kaiser 2006 [37]	mixed medical, neurological, trauma and surgical patients	MDT	GWDF	unidentifiable anatomy of the neck, short neck, cervical spine stiffness or trauma, coagulation disorders	controlled: SAPS II	intraoperative bleeding, desaturation, sc emphysema, pneumothorax, tube dislocation, technical difficulties, length of procedure	MDT: intensivist GWDF: intensivist	MDT: ICU GWDF: ICU	yes, in MDT and GWDF	median 7 vs. median 4
Fikkers 2011 [30]	NR	SSDT	GWDF	goiter, no palpable landmarks, distance cricoid to manubrium <3 cm at maximum extension, infection at the TT site, severe uncorrectable coagulopathy, PEEP >10 cmH_2O, ICP >20 cm H_2O, age <18 yrs., body weight <40 kg	controlled: SOFA	intraoperative bleeding, desaturation, pneumothorax, false route, technical difficulties, difficult dilatation, difficult insertion, postoperative bleeding, accidental decannulation, stoma infection, tracheal stenosis, LOS ICU, length of procedure	SSDT: intensivist GWDF: intensivist	SSDT: ICU GWDF: ICU	yes, in SSDT and GWDF	median 8 vs. median 10

TT = tracheotomy; ST = surgical tracheotomy; PT = percutaneous tracheotomy; MDT = multiple dilatation tracheotomy (Ciaglia); SSDT = single step dilatation tracheotomy (blue rhino); TLT = translaryngeal tracheotomy (Fantoni); RDT = rotational dilatation tracheotomy (percutwist); BDT = balloon dilatation tracheotomy (blue dolphin); GWDF = guide wire dilatation forceps (Griggs); APACHE = acute physiology and chronic heatlh evaluation score; SAPS = simplifited acute physiology score; SOFA = sequential organ failure assessment score; BMI = body mass index; NR = not reported; d = day/s; mo = months; wk/s = week/s; ICU = intensive care unit; ENT = ear, nose and throat (specialist); OR = operation room, LOS = length of stay; PEEP = positive endexspiratory pressure; FiO_2 = fraction of inspired oxygen; ICP = intracranail pressure; CPP = cerebral perfusion pressure
