# Supplementary material for: Percutaneous and surgical tracheostomy in critically ill adult patients: a meta-analysis
Source: Crit Care. 2014 Dec 19;18(6):544. doi: 10.1186/s13054-014-0544-7 (PMC4293819; doi:10.1186/s13054-014-0544-7)

Fig. 1 Forest plot of comparison PT vs. ST

Fig. 1 a) Outcome: Major intraprocedural bleeding

Study or Subgroup	PT Events	PT Total	ST Events	ST Total	Weight	Odds Ratio M-H, Random, 95% CI
**1.1.2 MDT vs. ST**						
crofts 1995	0	25	0	28		Not estimable
freeman 2001	1	40	0	40	23.1%	3.08 [0.12, 77.80]
friedman 1996	0	26	0	27		Not estimable
gysin 1999	0	35	0	35		Not estimable
hazard 1991	0	22	0	24		Not estimable
holdgaard 1998	0	30	2	30	25.1%	0.19 [0.01, 4.06]
melloni 2002	0	25	0	25		Not estimable
porter 1999	0	12	0	12		Not estimable
silvester 2006	3	100	0	100	26.7%	7.22 [0.37, 141.52]
wu 2003	0	41	0	42		Not estimable
**Subtotal (95% CI)**		**356**		**363**	**74.9%**	**1.62 [0.18, 14.50]**
Total events	4		2			
Heterogeneity: $Tau^2 = 1.26$; $Chi^2 = 3.01$, df = 2 (P = 0.22); $I^2 = 34\%$						
Test for overall effect: Z = 0.43 (P = 0.67)						
**1.1.3 SSDT vs. ST**						
tabaee 2005	0	29	0	14		Not estimable
**Subtotal (95% CI)**		**29**		**14**		**Not estimable**
Total events	0		0			
Heterogeneity: Not applicable						
Test for overall effect: Not applicable						
**1.1.4 GWDF vs. ST**						
heikkinen 2000	2	30	0	26	25.1%	4.65 [0.21, 101.37]
sustic 2002	0	8	0	8		Not estimable
**Subtotal (95% CI)**		**38**		**34**	**25.1%**	**4.65 [0.21, 101.37]**
Total events	2		0			
Heterogeneity: Not applicable						
Test for overall effect: Z = 0.98 (P = 0.33)						
**1.1.5 TLT vs. ST**						
antonelli 2005	0	67	0	72		Not estimable
**Subtotal (95% CI)**		**67**		**72**		**Not estimable**
Total events	0		0			
Heterogeneity: Not applicable						
Test for overall effect: Not applicable						
**Total (95% CI)**		**490**		**483**	**100.0%**	**2.12 [0.41, 10.84]**
Total events	6		2			
Heterogeneity: $Tau^2 = 0.29$; $Chi^2 = 3.35$, df = 3 (P = 0.34); $I^2 = 10\%$						
Test for overall effect: Z = 0.90 (P = 0.37)						
Test for subgroup differences: $Chi^2 = 0.30$, df = 1 (P = 0.59), $I^2 = 0\%$						

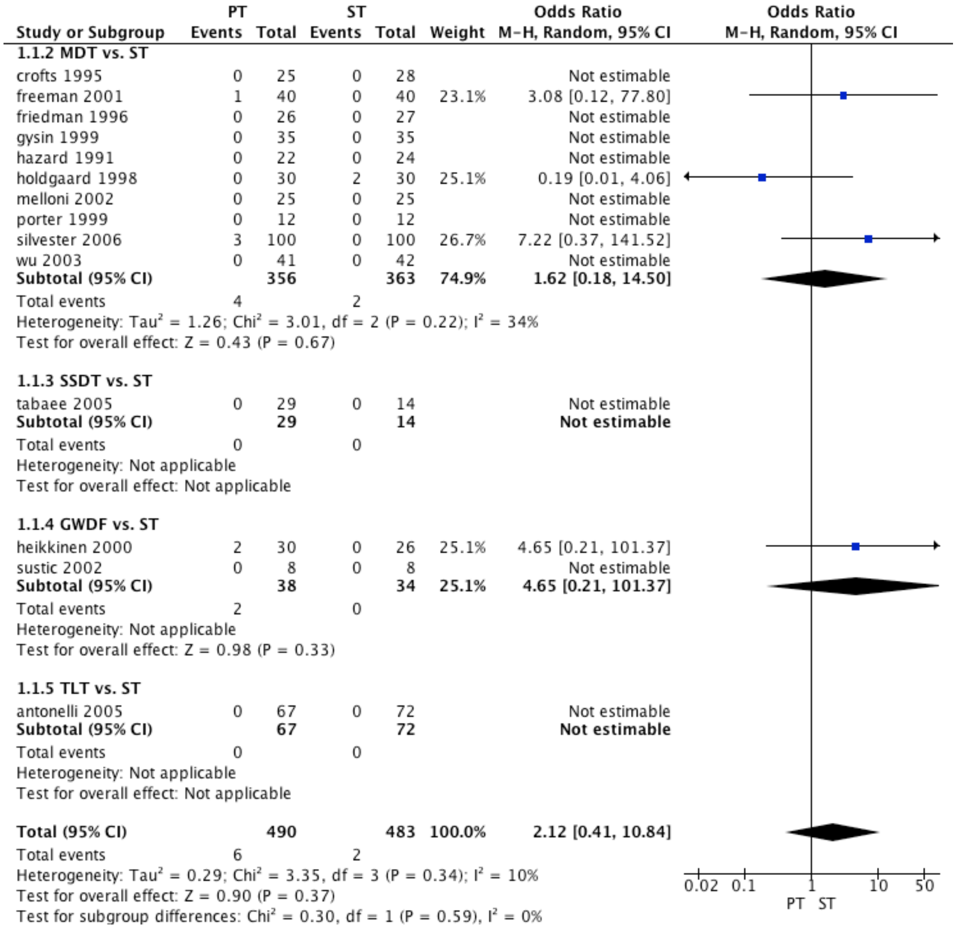

Fig. 1 b) Outcome: Tracheal stenosis

Study or Subgroup	PT Events	PT Total	ST Events	ST Total	Weight	Odds Ratio M-H, Fixed, 95% CI
**1.15.1 MDT vs. ST**						
gysin 1999	0	35	0	35		Not estimable
hazard 1991	2	22	5	24	53.1%	0.38 [0.07, 2.20]
melloni 2002	1	25	0	25	5.8%	3.12 [0.12, 80.39]
silvester 2006	0	100	0	100		Not estimable
wu 2003	0	41	1	42	17.9%	0.33 [0.01, 8.42]
**Subtotal (95% CI)**		**223**		**226**	**76.8%**	**0.57 [0.16, 2.08]**
Total events	3		6			
Heterogeneity: $Chi^2 = 1.37$, df = 2 (P = 0.51); $I^2 = 0\%$						
Test for overall effect: Z = 0.84 (P = 0.40)						
**1.15.2 SSDT vs. ST**						
**Subtotal (95% CI)**		**0**		**0**		**Not estimable**
Total events	0		0			
Heterogeneity: Not applicable						
Test for overall effect: Not applicable						
**1.15.3 GWDF vs. ST**						
**Subtotal (95% CI)**		**0**		**0**		**Not estimable**
Total events	0		0			
Heterogeneity: Not applicable						
Test for overall effect: Not applicable						
**1.15.4 TLT vs. ST**						
antonelli 2005	1	67	2	72	23.2%	0.53 [0.05, 5.99]
**Subtotal (95% CI)**		**67**		**72**	**23.2%**	**0.53 [0.05, 5.99]**
Total events	1		2			
Heterogeneity: Not applicable						
Test for overall effect: Z = 0.51 (P = 0.61)						
**Total (95% CI)**		**290**		**298**	**100.0%**	**0.56 [0.18, 1.76]**
Total events	4		8			
Heterogeneity: $Chi^2 = 1.37$, df = 3 (P = 0.71); $I^2 = 0\%$						
Test for overall effect: Z = 0.99 (P = 0.32)						
Test for subgroup differences: $Chi^2 = 0.00$, df = 1 (P = 0.95), $I^2 = 0\%$						

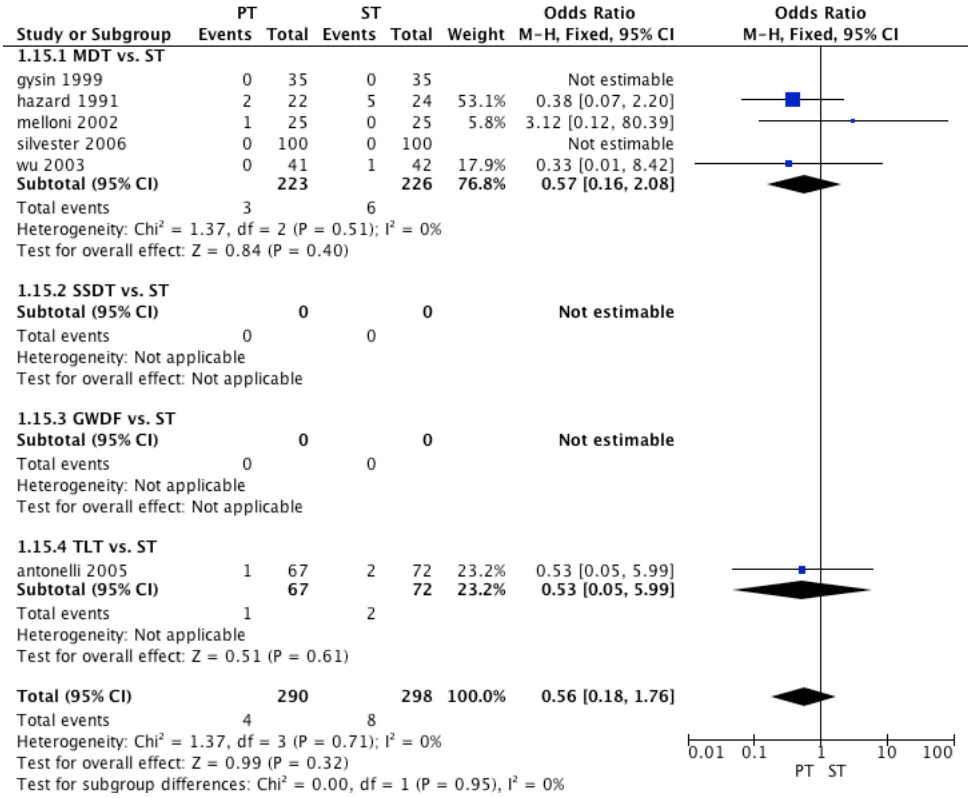

Fig. 1 c) Outcome: Hospital survival

Study or Subgroup	PT Events	PT Total	ST Events	ST Total	Weight	Odds Ratio M-H, Fixed, 95% CI
**1.17.1 MDT vs. ST**						
friedman 1996	16	26	18	27	11.2%	0.80 [0.26, 2.46]
hazard 1991	12	22	8	24	5.7%	2.40 [0.73, 7.92]
melloni 2002	21	25	18	25	4.7%	2.04 [0.51, 8.12]
silvester 2006	74	100	77	100	32.9%	0.85 [0.45, 1.62]
wu 2003	14	41	17	42	18.2%	0.76 [0.31, 1.86]
**Subtotal (95% CI)**		**214**		**218**	**72.7%**	**1.02 [0.67, 1.54]**
Total events	137		138			
Heterogeneity: Chi² = 3.84, df = 4 (P = 0.43); I² = 0%						
Test for overall effect: Z = 0.09 (P = 0.93)						
**1.17.2 SSDT vs. ST**						
**Subtotal (95% CI)**		**0**		**0**		**Not estimable**
Total events	0		0			
Heterogeneity: Not applicable						
Test for overall effect: Not applicable						
**1.17.3 GWDF vs. ST**						
**Subtotal (95% CI)**		**0**		**0**		**Not estimable**
Total events	0		0			
Heterogeneity: Not applicable						
Test for overall effect: Not applicable						
**1.17.4 TLT vs. ST**						
antonelli 2005	33	67	34	72	27.3%	1.08 [0.56, 2.11]
**Subtotal (95% CI)**		**67**		**72**	**27.3%**	**1.08 [0.56, 2.11]**
Total events	33		34			
Heterogeneity: Not applicable						
Test for overall effect: Z = 0.24 (P = 0.81)						
**Total (95% CI)**		**281**		**290**	**100.0%**	**1.04 [0.73, 1.48]**
Total events	170		172			
Heterogeneity: Chi² = 3.87, df = 5 (P = 0.57); I² = 0%						
Test for overall effect: Z = 0.21 (P = 0.84)						
Test for subgroup differences: Chi² = 0.02, df = 1 (P = 0.88), I² = 0%						

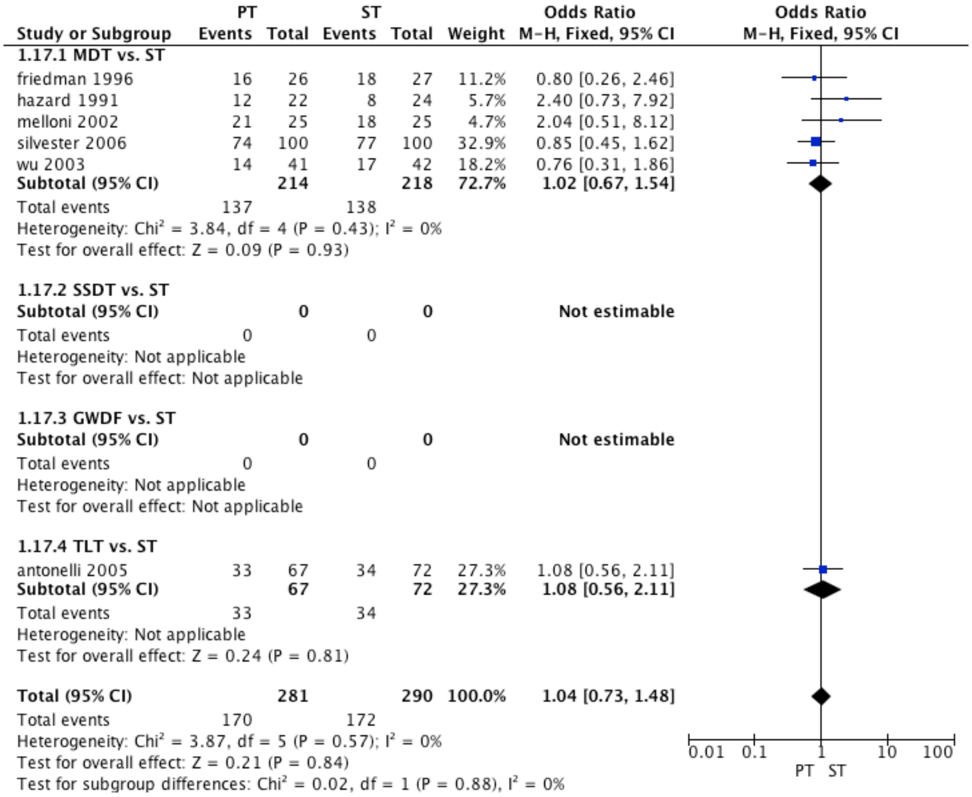

Fig. 1 d) Outcome: Duration of procedure

Study or Subgroup	PT Mean	PT SD	PT Total	ST Mean	ST SD	ST Total	Weight	Mean Difference IV, Random, 95% CI
**1.18.1 MDT vs. ST**								
freeman 2001	20.1	2	40	41.7	3.9	40	10.5%	−21.60 [−22.96, −20.24]
friedman 1996	8.2	4.9	26	33.9	14	27	9.7%	−25.70 [−31.31, −20.09]
gysin 1999	18.2	11.2	35	15.8	5.5	35	10.1%	2.40 [−1.73, 6.53]
hazard 1991	4.3	2.2	22	13.5	7.3	24	10.3%	−9.20 [−12.26, −6.14]
holdgaard 1998	0	0	0	0	0	0		Not estimable
melloni 2002	14	6	25	41	14	25	9.6%	−27.00 [−32.97, −21.03]
porter 1999	15.4	3.8	12	25.2	9.5	12	9.7%	−9.80 [−15.59, −4.01]
silvester 2006	20	0	100	17	0	100		Not estimable
wu 2003	22	12.1	41	41.5	5.9	42	10.1%	−19.50 [−23.61, −15.39]
**Subtotal (95% CI)**			**301**			**305**	**69.9%**	**−15.69 [−22.96, −8.43]**
Heterogeneity: Tau² = 90.80; Chi² = 176.35, df = 6 (P < 0.00001); I² = 97%								
Test for overall effect: Z = 4.23 (P < 0.0001)								
**1.18.2 SSDT vs. ST**								
tabaee 2005	8	0	29	236	0	14		Not estimable
**Subtotal (95% CI)**			**29**			**14**		**Not estimable**
Heterogeneity: Not applicable								
Test for overall effect: Not applicable								
**1.18.3 GWDF vs. ST**								
heikkinen 2000	11.13	6.4	30	14.4	6	26	10.2%	−3.27 [−6.52, −0.02]
sustic 2002	8	6	8	21	7	8	9.5%	−13.00 [−19.39, −6.61]
**Subtotal (95% CI)**			**38**			**34**	**19.7%**	**−7.73 [−17.23, 1.77]**
Heterogeneity: Tau² = 40.65; Chi² = 7.08, df = 1 (P = 0.008); I² = 86%								
Test for overall effect: Z = 1.59 (P = 0.11)								
**1.18.4 TLT vs. ST**								
antonelli 2005	17	10	67	22	6	72	10.3%	−5.00 [−7.77, −2.23]
**Subtotal (95% CI)**			**67**			**72**	**10.3%**	**−5.00 [−7.77, −2.23]**
Heterogeneity: Not applicable								
Test for overall effect: Z = 3.54 (P = 0.0004)								
**Total (95% CI)**			**435**			**425**	**100.0%**	**−13.06 [−19.37, −6.76]**
Heterogeneity: Tau² = 98.33; Chi² = 310.12, df = 9 (P < 0.00001); I² = 97%								
Test for overall effect: Z = 4.06 (P < 0.0001)								
Test for subgroup differences: Chi² = 7.35, df = 2 (P = 0.03), I² = 72.8%								

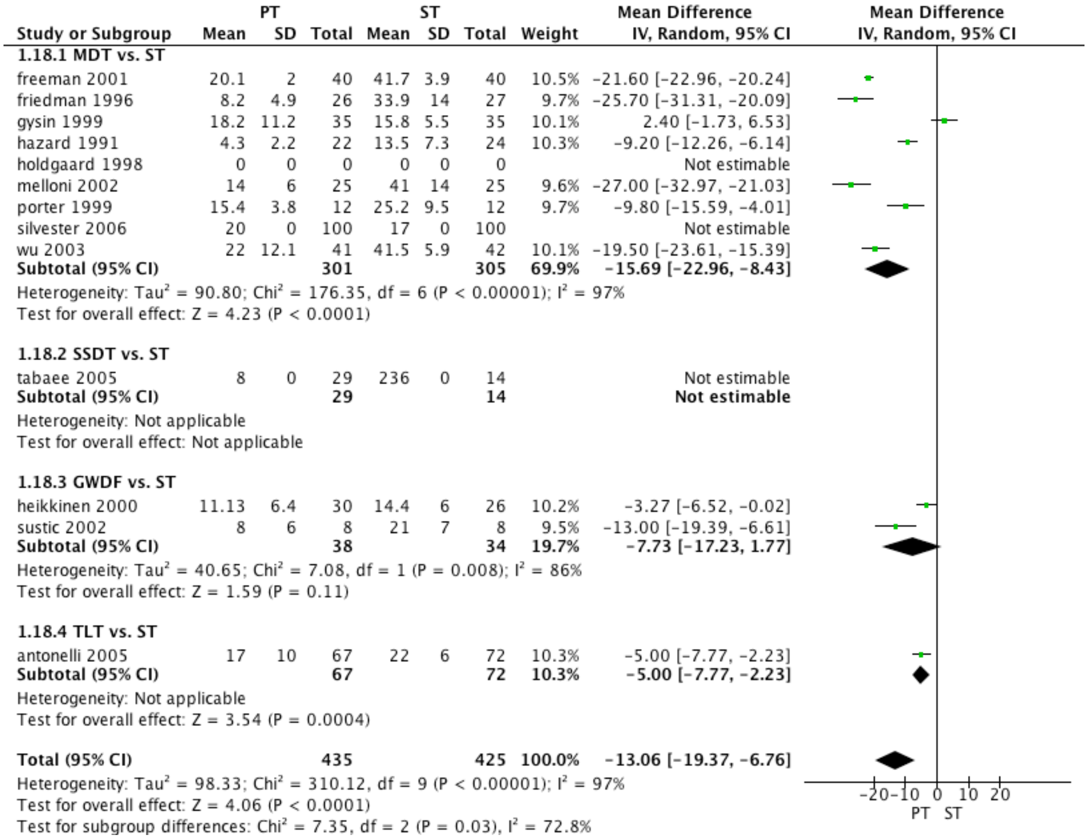

Fig 1 e) Outcome: Minor postprocedural bleeding

Study or Subgroup	PT Events	PT Total	ST Events	ST Total	Weight	Odds Ratio M-H, Fixed, 95% CI
**1.11.1 MDT vs. ST**						
friedman 1996	1	26	3	27	10.8%	0.32 [0.03, 3.29]
gysin 1999	4	35	4	35	13.5%	1.00 [0.23, 4.36]
hazard 1991	1	22	4	24	14.0%	0.24 [0.02, 2.32]
holdgaard 1998	2	30	9	30	32.1%	0.17 [0.03, 0.85]
melloni 2002	1	25	1	25	3.7%	1.00 [0.06, 16.93]
porter 1999	0	12	0	12		Not estimable
silvester 2006	4	100	1	100	3.7%	4.13 [0.45, 37.57]
wu 2003	2	41	3	42	10.8%	0.67 [0.11, 4.21]
**Subtotal (95% CI)**		**291**		**295**	**88.5%**	**0.58 [0.30, 1.13]**
Total events	15		25			
Heterogeneity: $Chi^2 = 6.80$, df = 6 (P = 0.34); $I^2 = 12\%$						
Test for overall effect: Z = 1.59 (P = 0.11)						
**1.11.2 SSDT vs. ST**						
tabaee 2005	1	29	0	14	2.4%	1.53 [0.06, 39.86]
**Subtotal (95% CI)**		**29**		**14**	**2.4%**	**1.53 [0.06, 39.86]**
Total events	1		0			
Heterogeneity: Not applicable						
Test for overall effect: Z = 0.25 (P = 0.80)						
**1.11.3 GWDF vs. ST**						
heikkinen 2000	1	30	0	26	1.9%	2.69 [0.11, 69.04]
sustic 2002	0	8	0	8		Not estimable
**Subtotal (95% CI)**		**38**		**34**	**1.9%**	**2.69 [0.11, 69.04]**
Total events	1		0			
Heterogeneity: Not applicable						
Test for overall effect: Z = 0.60 (P = 0.55)						
**1.11.4 TLT vs. ST**						
antonelli 2005	2	67	2	72	7.1%	1.08 [0.15, 7.87]
**Subtotal (95% CI)**		**67**		**72**	**7.1%**	**1.08 [0.15, 7.87]**
Total events	2		2			
Heterogeneity: Not applicable						
Test for overall effect: Z = 0.07 (P = 0.94)						
**Total (95% CI)**		**425**		**415**	**100.0%**	**0.68 [0.38, 1.24]**
Total events	19		27			
Heterogeneity: $Chi^2 = 8.09$, df = 9 (P = 0.52); $I^2 = 0\%$						
Test for overall effect: Z = 1.25 (P = 0.21)						
Test for subgroup differences: $Chi^2 = 1.34$, df = 3 (P = 0.72), $I^2 = 0\%$						

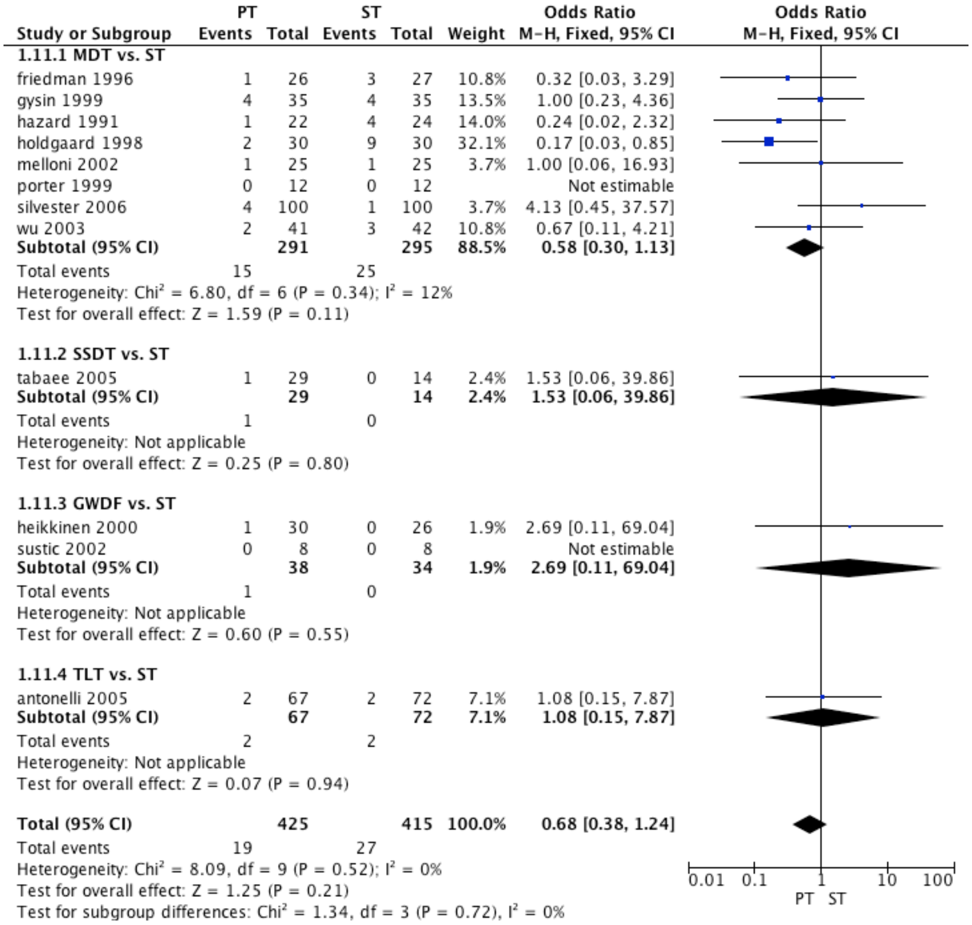

Fig. 1 f) Outcome: Minor intraprocedural bleeding

Study or Subgroup	PT Events	PT Total	ST Events	ST Total	Weight	Odds Ratio M-H, Random, 95% CI
**1.3.1 MDT vs. ST**						
crofts 1995	3	25	3	28	12.8%	1.14 [0.21, 6.22]
freeman 2001	0	40	0	40		Not estimable
friedman 1996	3	26	3	27	12.8%	1.04 [0.19, 5.71]
gysin 1999	0	35	0	35		Not estimable
hazard 1991	0	22	0	24		Not estimable
holdgaard 1998	6	30	24	30	15.4%	0.06 [0.02, 0.22]
melloni 2002	2	25	0	25	6.8%	5.43 [0.25, 118.96]
silvester 2006	1	100	1	100	7.8%	1.00 [0.06, 16.21]
wu 2003	1	41	1	42	7.7%	1.02 [0.06, 16.95]
**Subtotal (95% CI)**		**344**		**351**	**63.3%**	**0.68 [0.16, 2.84]**
Total events	16		32			
Heterogeneity: $Tau^2 = 1.94$; $Chi^2 = 14.53$, df = 5 (P = 0.01); $I^2 = 66\%$						
Test for overall effect: Z = 0.53 (P = 0.60)						
**1.3.2 SSDT vs. ST**						
tabaee 2005	3	29	5	14	13.2%	0.21 [0.04, 1.05]
**Subtotal (95% CI)**		**29**		**14**	**13.2%**	**0.21 [0.04, 1.05]**
Total events	3		5			
Heterogeneity: Not applicable						
Test for overall effect: Z = 1.90 (P = 0.06)						
**1.3.3 GWDF vs. ST**						
heikkinen 2000	3	30	0	26	7.1%	6.75 [0.33, 136.96]
sustic 2002	1	8	1	8	7.2%	1.00 [0.05, 19.36]
**Subtotal (95% CI)**		**38**		**34**	**14.3%**	**2.56 [0.31, 21.14]**
Total events	4		1			
Heterogeneity: $Tau^2 = 0.00$; $Chi^2 = 0.81$, df = 1 (P = 0.37); $I^2 = 0\%$						
Test for overall effect: Z = 0.87 (P = 0.38)						
**1.3.4 TLT vs. ST**						
antonelli 2005	1	67	2	72	9.2%	0.53 [0.05, 5.99]
**Subtotal (95% CI)**		**67**		**72**	**9.2%**	**0.53 [0.05, 5.99]**
Total events	1		2			
Heterogeneity: Not applicable						
Test for overall effect: Z = 0.51 (P = 0.61)						
**Total (95% CI)**		**478**		**471**	**100.0%**	**0.66 [0.25, 1.77]**
Total events	24		40			
Heterogeneity: $Tau^2 = 1.23$; $Chi^2 = 18.93$, df = 9 (P = 0.03); $I^2 = 52\%$						
Test for overall effect: Z = 0.83 (P = 0.41)						
Test for subgroup differences: $Chi^2 = 3.50$, df = 3 (P = 0.32), $I^2 = 14.2\%$						

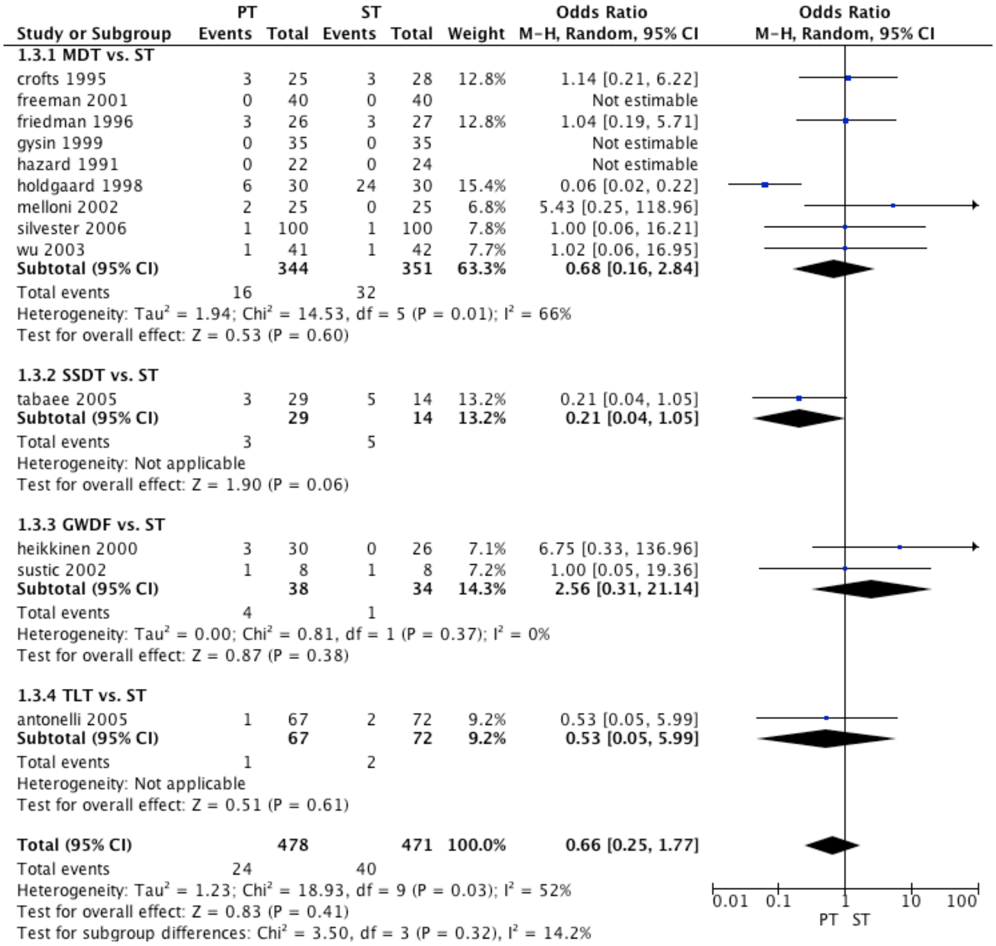

Fig. 1 g) Outcome: False route

Study or Subgroup	PT Events	PT Total	ST Events	ST Total	Weight	Odds Ratio M-H, Fixed, 95% CI
**1.8.1 MDT vs. ST**						
friedman 1996	1	26	0	27	16.2%	3.24 [0.13, 83.08]
gysin 1999	4	35	2	35	61.8%	2.13 [0.36, 12.46]
holdgaard 1998	0	30	0	30		Not estimable
melloni 2002	0	25	0	25		Not estimable
silvester 2006	0	100	0	100		Not estimable
**Subtotal (95% CI)**		**216**		**217**	**77.9%**	**2.36 [0.50, 11.07]**
Total events	5		2			
Heterogeneity: $Chi^2 = 0.05$, df = 1 (P = 0.82); $I^2 = 0\%$						
Test for overall effect: Z = 1.09 (P = 0.28)						
**1.8.2 SSDT vs. ST**						
tabaee 2005	1	29	0	14	22.1%	1.53 [0.06, 39.86]
**Subtotal (95% CI)**		**29**		**14**	**22.1%**	**1.53 [0.06, 39.86]**
Total events	1		0			
Heterogeneity: Not applicable						
Test for overall effect: Z = 0.25 (P = 0.80)						
**1.8.3 GWDF vs. ST**						
**Subtotal (95% CI)**		**0**		**0**		**Not estimable**
Total events	0		0			
Heterogeneity: Not applicable						
Test for overall effect: Not applicable						
**1.8.4 TLT vs. ST**						
**Subtotal (95% CI)**		**0**		**0**		**Not estimable**
Total events	0		0			
Heterogeneity: Not applicable						
Test for overall effect: Not applicable						
**Total (95% CI)**		**245**		**231**	**100.0%**	**2.17 [0.54, 8.82]**
Total events	6		2			
Heterogeneity: $Chi^2 = 0.10$, df = 2 (P = 0.95); $I^2 = 0\%$						
Test for overall effect: Z = 1.09 (P = 0.28)						
Test for subgroup differences: $Chi^2 = 0.06$, df = 1 (P = 0.81), $I^2 = 0\%$						

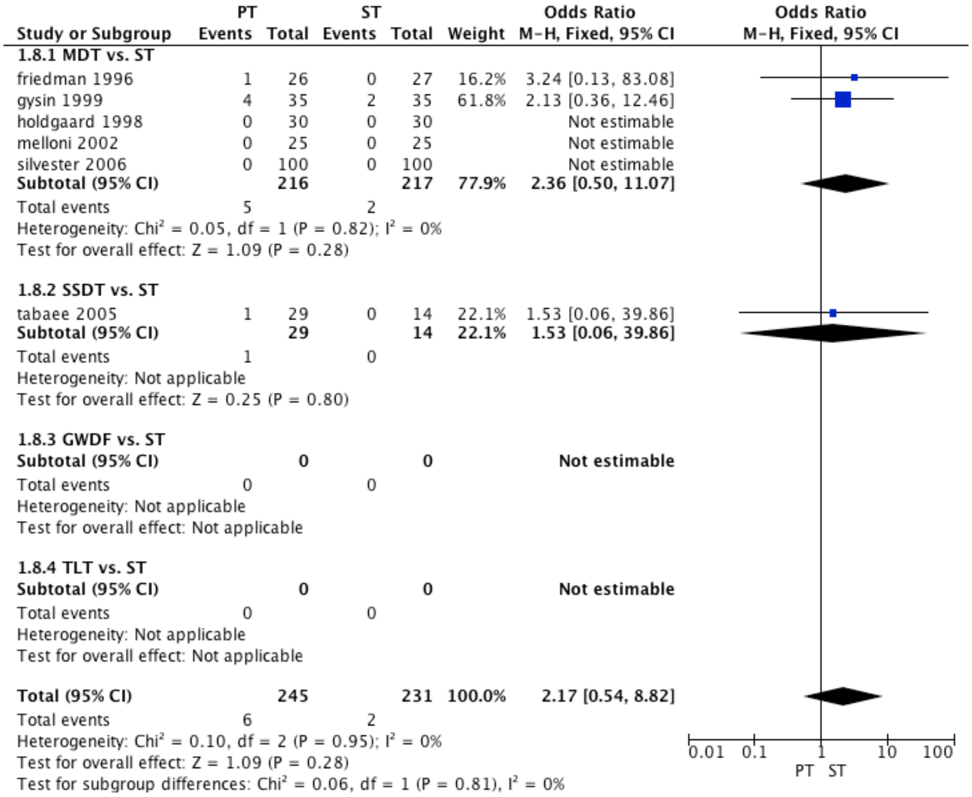

Fig. 1 h) Outcome: Subcutaneous emphysema

Study or Subgroup	PT Events	PT Total	ST Events	ST Total	Weight	Odds Ratio M-H, Fixed, 95% CI
**1.5.1 MDT vs. ST**						
crofts 1995	0	25	1	28	32.6%	0.36 [0.01, 9.23]
friedman 1996	0	26	1	27	33.8%	0.33 [0.01, 8.56]
gysin 1999	0	35	0	35		Not estimable
holdgaard 1998	0	30	0	30		Not estimable
**Subtotal (95% CI)**		**116**		**120**	**66.4%**	**0.35 [0.03, 3.44]**
Total events	0		2			
Heterogeneity: Chi² = 0.00, df = 1 (P = 0.97); I² = 0%						
Test for overall effect: Z = 0.91 (P = 0.36)						
**1.5.2 SSDT vs. ST**						
**Subtotal (95% CI)**		**0**		**0**		**Not estimable**
Total events	0		0			
Heterogeneity: Not applicable						
Test for overall effect: Not applicable						
**1.5.3 GWDF vs. ST**						
**Subtotal (95% CI)**		**0**		**0**		**Not estimable**
Total events	0		0			
Heterogeneity: Not applicable						
Test for overall effect: Not applicable						
**1.5.4 TLT vs. ST**						
antonelli 2005	0	67	1	72	33.6%	0.35 [0.01, 8.82]
**Subtotal (95% CI)**		**67**		**72**	**33.6%**	**0.35 [0.01, 8.82]**
Total events	0		1			
Heterogeneity: Not applicable						
Test for overall effect: Z = 0.63 (P = 0.53)						
**Total (95% CI)**		**183**		**192**	**100.0%**	**0.35 [0.05, 2.26]**
Total events	0		3			
Heterogeneity: Chi² = 0.00, df = 2 (P = 1.00); I² = 0%						
Test for overall effect: Z = 1.11 (P = 0.27)						
Test for subgroup differences: Chi² = 0.00, df = 1 (P = 0.99), I² = 0%						

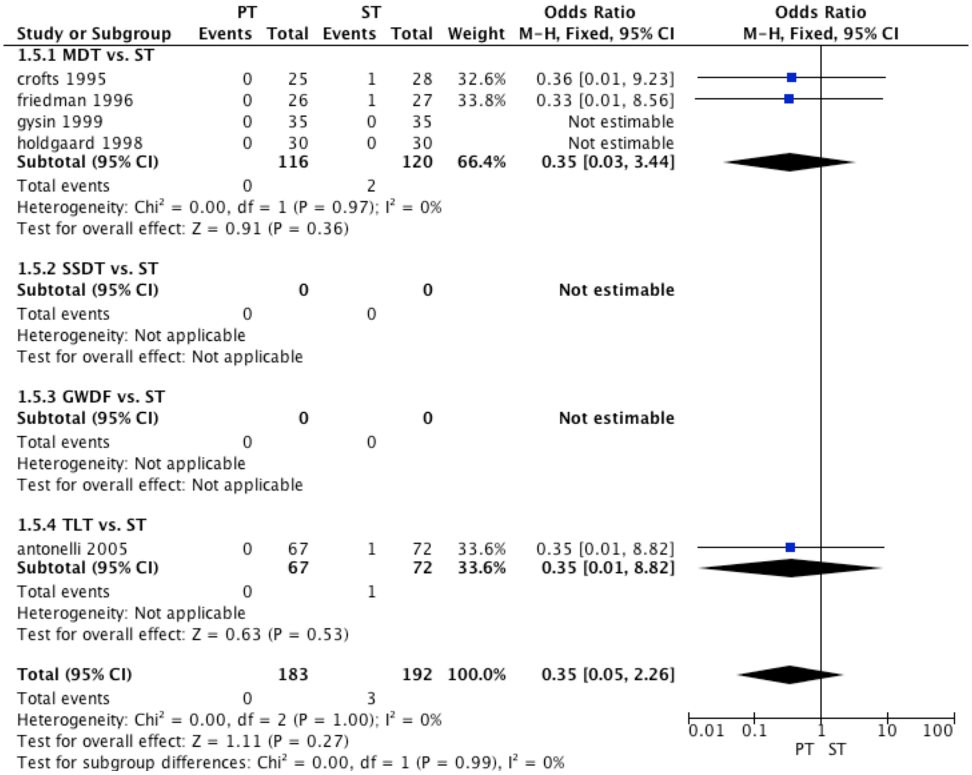

Fig. 1 i) Outcome: Pneumothorax

Study or Subgroup	PT Events	PT Total	ST Events	ST Total	Weight	Odds Ratio M-H, Fixed, 95% CI
**1.6.1 MDT vs. ST**						
crofts 1995	0	25	1	28	49.7%	0.36 [0.01, 9.23]
gysin 1999	0	35	0	35		Not estimable
hazard 1991	1	22	1	24	32.6%	1.10 [0.06, 18.64]
holdgaard 1998	0	30	0	30		Not estimable
porter 1999	0	12	0	12		Not estimable
silvester 2006	1	100	0	100	17.6%	3.03 [0.12, 75.28]
**Subtotal (95% CI)**		**224**		**229**	**100.0%**	**1.07 [0.21, 5.46]**
Total events	2		2			
Heterogeneity: Chi² = 0.84, df = 2 (P = 0.66); I² = 0%						
Test for overall effect: Z = 0.08 (P = 0.94)						
**1.6.2 SSDT vs. ST**						
**Subtotal (95% CI)**		**0**		**0**		**Not estimable**
Total events	0		0			
Heterogeneity: Not applicable						
Test for overall effect: Not applicable						
**1.6.3 GWDF vs. ST**						
**Subtotal (95% CI)**		**0**		**0**		**Not estimable**
Total events	0		0			
Heterogeneity: Not applicable						
Test for overall effect: Not applicable						
**1.6.4 TLT vs. ST**						
**Subtotal (95% CI)**		**0**		**0**		**Not estimable**
Total events	0		0			
Heterogeneity: Not applicable						
Test for overall effect: Not applicable						
**Total (95% CI)**		**224**		**229**	**100.0%**	**1.07 [0.21, 5.46]**
Total events	2		2			
Heterogeneity: Chi² = 0.84, df = 2 (P = 0.66); I² = 0%						
Test for overall effect: Z = 0.08 (P = 0.94)						
Test for subgroup differences: Not applicable						

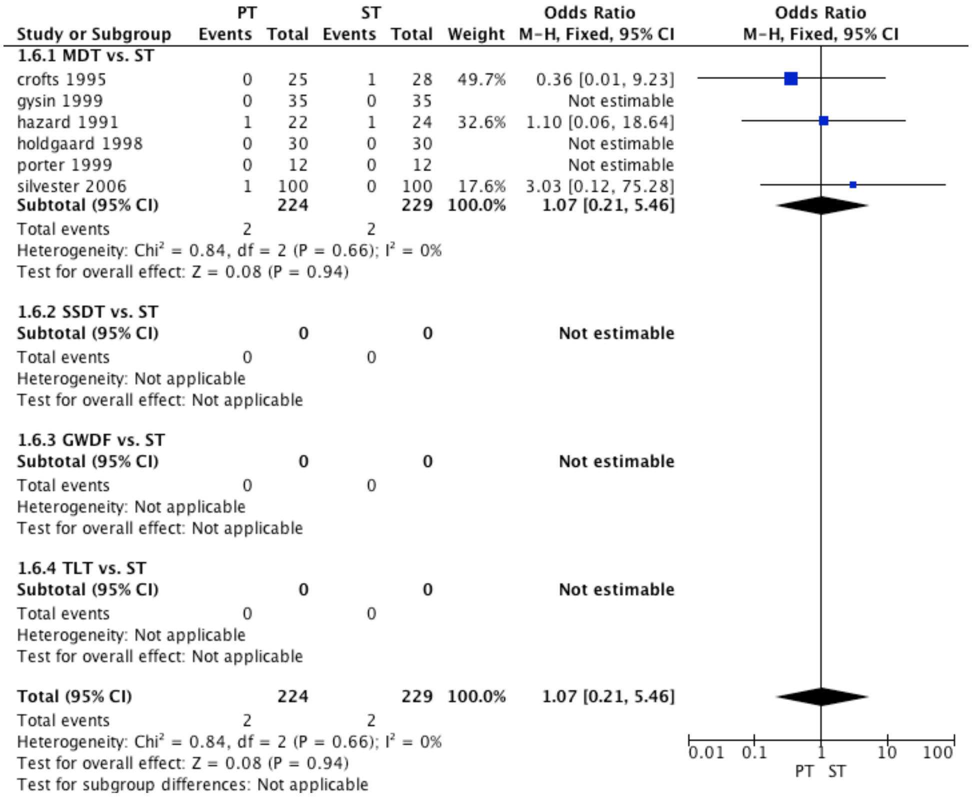

Fig. 1 j) Outcome: Desaturation

Study or Subgroup	PT Events	PT Total	ST Events	ST Total	Weight	Odds Ratio M-H, Random, 95% CI
**1.4.1 MDT vs. ST**						
friedman 1996	0	26	3	27	17.6%	0.13 [0.01, 2.69]
gysin 1999	0	35	0	35		Not estimable
holdgaard 1998	0	30	0	30		Not estimable
porter 1999	3	12	1	12	27.1%	3.67 [0.32, 41.59]
silvester 2006	0	100	0	100		Not estimable
**Subtotal (95% CI)**		**203**		**204**	**44.6%**	**0.79 [0.03, 21.00]**
Total events	3		4			
Heterogeneity: $Tau^2 = 3.70$; $Chi^2 = 2.90$, df = 1 (P = 0.09); $I^2 = 65\%$						
Test for overall effect: Z = 0.14 (P = 0.89)						
**1.4.2 SSDT vs. ST**						
tabaee 2005	1	29	0	14	15.0%	1.53 [0.06, 39.86]
**Subtotal (95% CI)**		**29**		**14**	**15.0%**	**1.53 [0.06, 39.86]**
Total events	1		0			
Heterogeneity: Not applicable						
Test for overall effect: Z = 0.25 (P = 0.80)						
**1.4.3 GWDF vs. ST**						
sustic 2002	0	8	0	8		Not estimable
**Subtotal (95% CI)**		**8**		**8**		**Not estimable**
Total events	0		0			
Heterogeneity: Not applicable						
Test for overall effect: Not applicable						
**1.4.4 TLT vs. ST**						
antonelli 2005	2	67	2	72	40.4%	1.08 [0.15, 7.87]
**Subtotal (95% CI)**		**67**		**72**	**40.4%**	**1.08 [0.15, 7.87]**
Total events	2		2			
Heterogeneity: Not applicable						
Test for overall effect: Z = 0.07 (P = 0.94)						
**Total (95% CI)**		**307**		**298**	**100.0%**	**1.09 [0.31, 3.87]**
Total events	6		6			
Heterogeneity: $Tau^2 = 0.00$; $Chi^2 = 2.94$, df = 3 (P = 0.40); $I^2 = 0\%$						
Test for overall effect: Z = 0.14 (P = 0.89)						
Test for subgroup differences: $Chi^2 = 0.08$, df = 2 (P = 0.96), $I^2 = 0\%$						

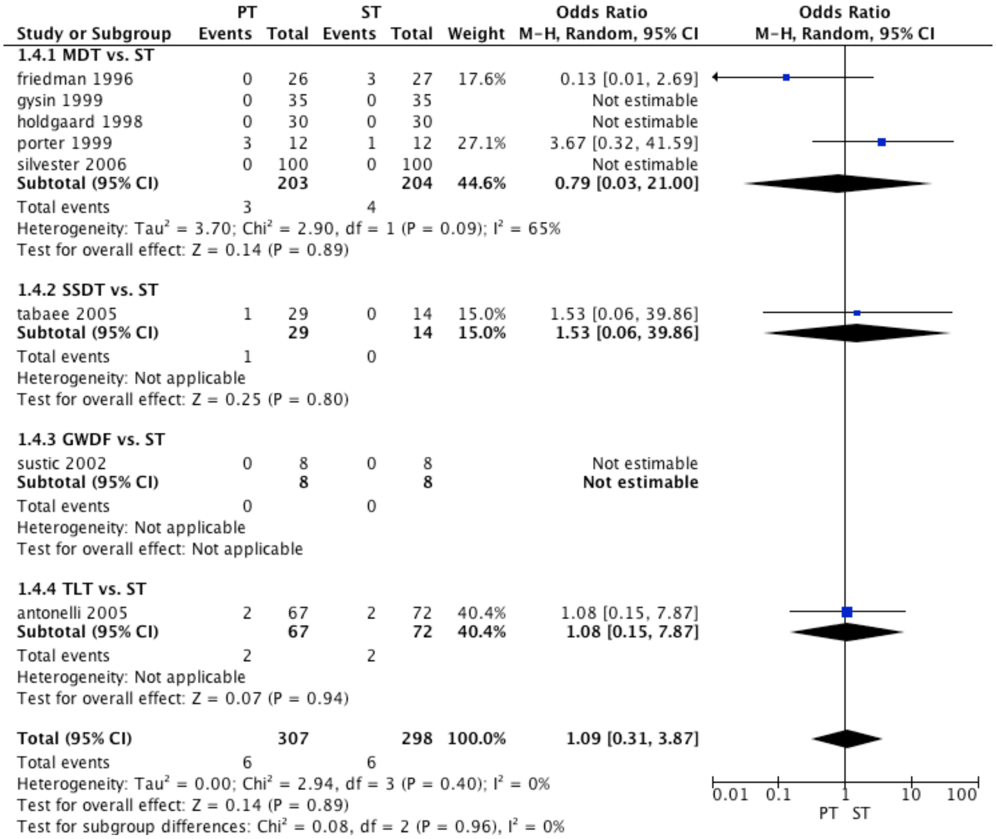

Fig. 2 Forest plot of comparison MDT/ SSDT vs. RDT/ BDT/ GWDF

Fig. 2 a) Outcome: Major intraprocedural bleeding

Study or Subgroup	MDT / SSDT Events	MDT / SSDT Total	RDT/ BDT/GWDF Events	RDT/ BDT/GWDF Total	Weight	Odds Ratio M-H, Fixed, 95% CI
**2.1.1 MDT/ SSDT vs. GWDF**						
ambesh 2002	1	30	5	30	33.9%	0.17 [0.02, 1.58]
anon 2004	0	27	0	26		Not estimable
fikkers 2011	1	60	3	60	20.7%	0.32 [0.03, 3.19]
kaiser 2006	0	48	0	52		Not estimable
nates 2000	0	52	3	48	25.3%	0.12 [0.01, 2.46]
van heurn 2001	2	63	3	64	20.2%	0.67 [0.11, 4.13]
**Subtotal (95% CI)**		**280**		**280**	**100.0%**	**0.29 [0.10, 0.85]**
Total events	4		14			
Heterogeneity: Chi² = 1.33, df = 3 (P = 0.72); I² = 0%						
Test for overall effect: Z = 2.25 (P = 0.02)						
**2.1.2 MDT/ SSDT vs. RDT/ BDT**						
byhahn 2002	0	35	0	35		Not estimable
cianchi 2010	0	35	0	35		Not estimable
**Subtotal (95% CI)**		**70**		**70**		**Not estimable**
Total events	0		0			
Heterogeneity: Not applicable						
Test for overall effect: Not applicable						
**Total (95% CI)**		**350**		**350**	**100.0%**	**0.29 [0.10, 0.85]**
Total events	4		14			
Heterogeneity: Chi² = 1.33, df = 3 (P = 0.72); I² = 0%						
Test for overall effect: Z = 2.25 (P = 0.02)						
Test for subgroup differences: Not applicable						

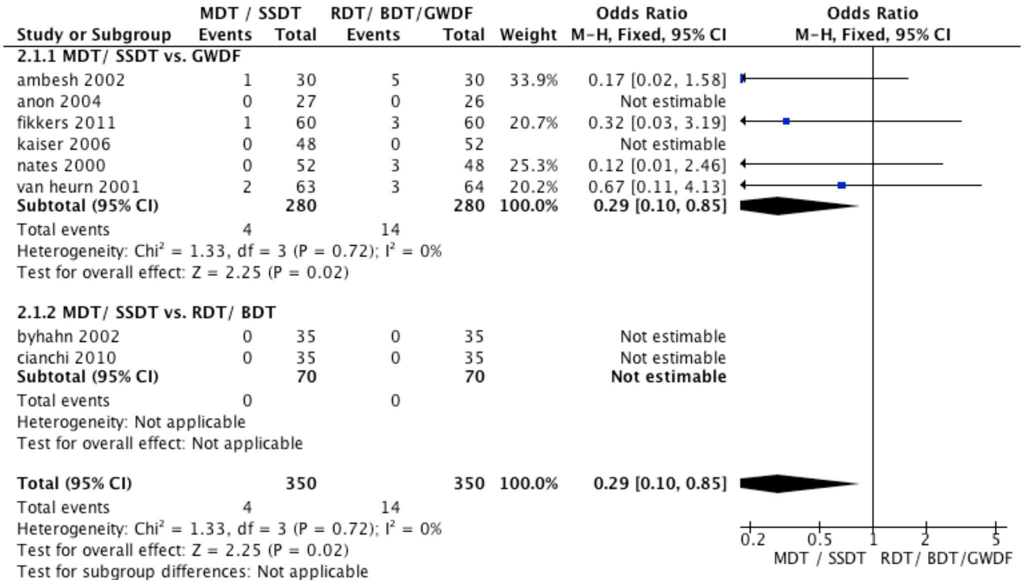

Fig. 2 b) Outcome: Tracheal stenosis

Study or Subgroup	MDT / SSDT Events	MDT / SSDT Total	RDT/ BDT/GWDF Events	RDT/ BDT/GWDF Total	Weight	Odds Ratio M-H, Fixed, 95% CI
**2.15.1 MDT/ SSDT vs. GWDF**						
anon 2004	0	27	0	26		Not estimable
fikkers 2011	0	60	1	60	75.6%	0.33 [0.01, 8.21]
**Subtotal (95% CI)**		**87**		**86**	**75.6%**	**0.33 [0.01, 8.21]**
Total events	0		1			
Heterogeneity: Not applicable						
Test for overall effect: Z = 0.68 (P = 0.50)						
**2.15.2 MDT/ SSDT vs. RDT/ BDT**						
cianchi 2010	1	35	0	35	24.4%	3.09 [0.12, 78.41]
**Subtotal (95% CI)**		**35**		**35**	**24.4%**	**3.09 [0.12, 78.41]**
Total events	1		0			
Heterogeneity: Not applicable						
Test for overall effect: Z = 0.68 (P = 0.49)						
**Total (95% CI)**		**122**		**121**	**100.0%**	**1.00 [0.14, 7.22]**
Total events	1		1			
Heterogeneity: Chi² = 0.93, df = 1 (P = 0.34); I² = 0%						
Test for overall effect: Z = 0.00 (P = 1.00)						
Test for subgroup differences: Chi² = 0.93, df = 1 (P = 0.34), I² = 0%						

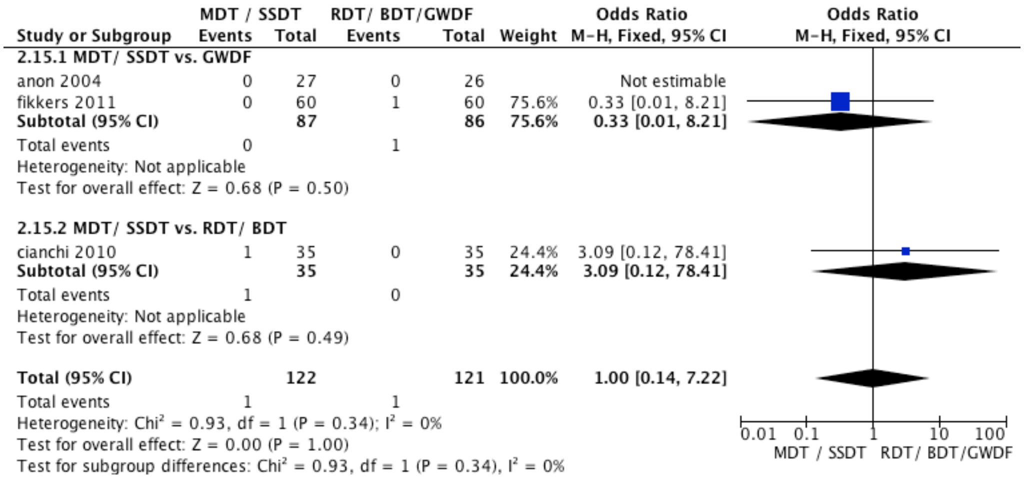

Fig. 2 c) Outcome: Hospital survival

Study or Subgroup	MDT / SSDT Events	MDT / SSDT Total	RDT/ BDT/GWDF Events	RDT/ BDT/GWDF Total	Weight	Odds Ratio M-H, Random, 95% CI
**2.17.1 MDT/ SSDT vs. GWDF**						
van heurn 2001	40	63	46	64	60.3%	0.68 [0.32, 1.44]
**Subtotal (95% CI)**		**63**		**64**	**60.3%**	**0.68 [0.32, 1.44]**
Total events	40		46			
Heterogeneity: Not applicable						
Test for overall effect: Z = 1.01 (P = 0.31)						
**2.17.2 MDT/ SSDT vs. RDT/ BDT**						
cianchi 2010	26	35	23	35	39.7%	1.51 [0.54, 4.22]
**Subtotal (95% CI)**		**35**		**35**	**39.7%**	**1.51 [0.54, 4.22]**
Total events	26		23			
Heterogeneity: Not applicable						
Test for overall effect: Z = 0.78 (P = 0.44)						
**Total (95% CI)**		**98**		**99**	**100.0%**	**0.93 [0.44, 2.00]**
Total events	66		69			
Heterogeneity: Tau² = 0.11; Chi² = 1.50, df = 1 (P = 0.22); I² = 33%						
Test for overall effect: Z = 0.18 (P = 0.86)						
Test for subgroup differences: Chi² = 1.50, df = 1 (P = 0.22), I² = 33.2%						

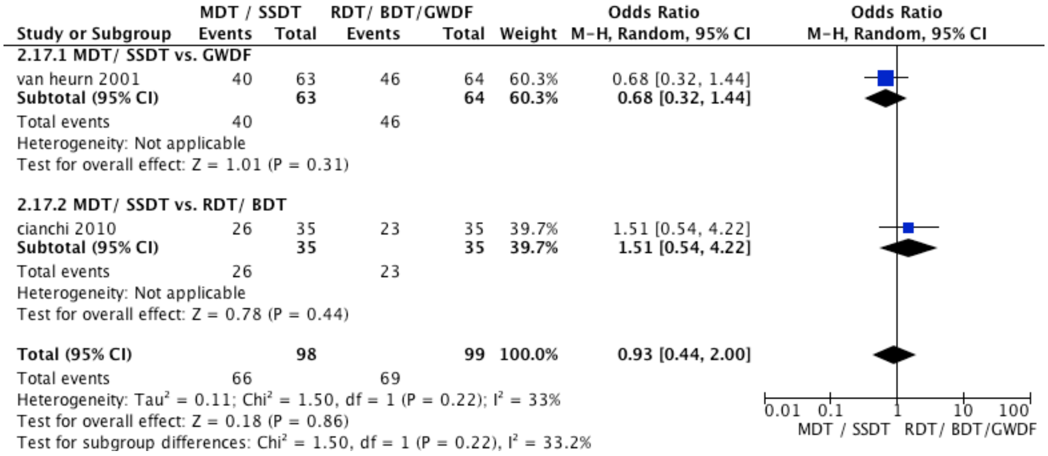

Fig. 2 d) Outcome: Duration of procedure

Study or Subgroup	MDT / SSDT Mean	SD	Total	RDT/ BDT/GWDF Mean	SD	Total	Weight	Mean Difference IV, Fixed, 95% CI
**2.18.1 MDT/ SSDT vs. GWDF**								
ambesh 2002	7.5	2.5	30	6.5	4.5	30	36.0%	1.00 [−0.84, 2.84]
nates 2000	9.3	7	52	10	12	48	8.1%	−0.70 [−4.59, 3.19]
van heurn 2001	7	3.5	63	6.4	4.9	64	55.9%	0.60 [−0.88, 2.08]
**Subtotal (95% CI)**			**145**			**142**	**100.0%**	**0.64 [−0.47, 1.75]**
Heterogeneity: Chi² = 0.60, df = 2 (P = 0.74); I² = 0%								
Test for overall effect: Z = 1.13 (P = 0.26)								
**2.18.2 MDT /SSDT vs. RDT/ BDT**								
**Subtotal (95% CI)**			**0**			**0**		**Not estimable**
Heterogeneity: Not applicable								
Test for overall effect: Not applicable								
**Total (95% CI)**			**145**			**142**	**100.0%**	**0.64 [−0.47, 1.75]**
Heterogeneity: Chi² = 0.60, df = 2 (P = 0.74); I² = 0%								
Test for overall effect: Z = 1.13 (P = 0.26)								
Test for subgroup differences: Not applicable								

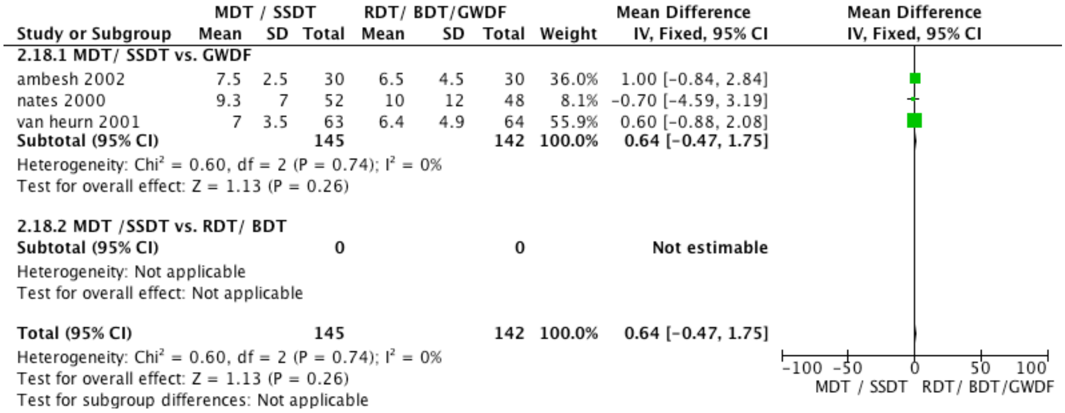

Fig 2 e) Outcome: Minor postprocedural bleeding

Study or Subgroup	MDT / SSDT Events	Total	RDT/ BDT/GWDF Events	Total	Weight	Odds Ratio M-H, Random, 95% CI
**2.11.1 MDT/ SSDT vs. GWDF**						
ambesh 2002	0	30	0	30		Not estimable
fikkers 2011	8	60	4	60	48.4%	2.15 [0.61, 7.58]
**Subtotal (95% CI)**		**90**		**90**	**48.4%**	**2.15 [0.61, 7.58]**
Total events	8		4			
Heterogeneity: Not applicable						
Test for overall effect: Z = 1.20 (P = 0.23)						
**2.11.2 MDT/ SSDT vs. RDT/ BDT / GWDF**						
cianchi 2010	12	35	24	35	51.6%	0.24 [0.09, 0.65]
**Subtotal (95% CI)**		**35**		**35**	**51.6%**	**0.24 [0.09, 0.65]**
Total events	12		24			
Heterogeneity: Not applicable						
Test for overall effect: Z = 2.81 (P = 0.005)						
**Total (95% CI)**		**125**		**125**	**100.0%**	**0.69 [0.08, 5.98]**
Total events	20		28			
Heterogeneity: Tau² = 2.08; Chi² = 7.21, df = 1 (P = 0.007); I² = 86%						
Test for overall effect: Z = 0.33 (P = 0.74)						
Test for subgroup differences: Chi² = 7.19, df = 1 (P = 0.007), I² = 86.1%						

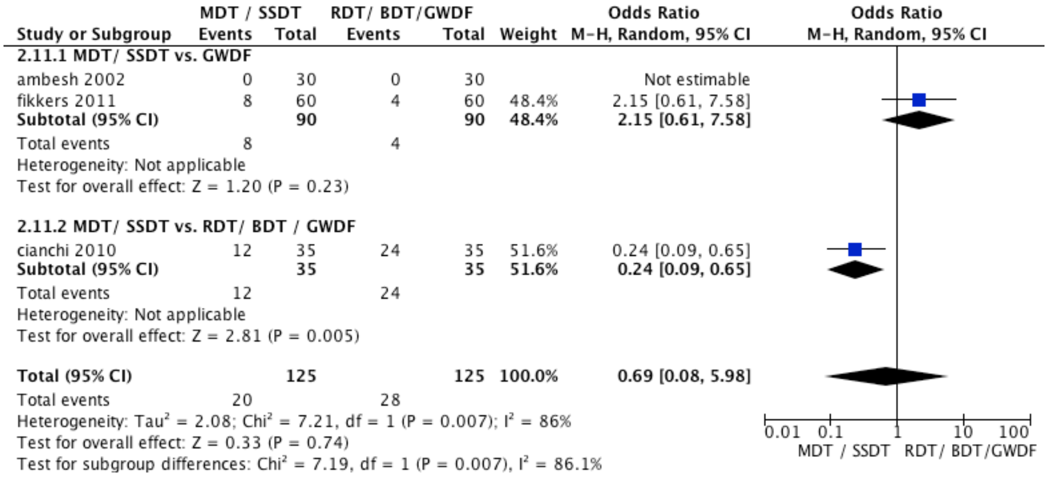

Fig. 2 f) Outcome: Minor intraprocedural bleeding

Study or Subgroup	MDT / SSDT Events	Total	RDT/ BDT/GWDF Events	Total	Weight	Odds Ratio M-H, Random, 95% CI
**2.3.1 MDT/ SSDT vs. GWDF**						
anon 2004	2	27	1	26	11.2%	2.00 [0.17, 23.49]
fikkers 2011	7	60	11	60	27.6%	0.59 [0.21, 1.64]
kaiser 2006	17	48	6	52	27.4%	4.20 [1.49, 11.85]
van heurn 2001	2	63	4	64	17.6%	0.49 [0.09, 2.79]
**Subtotal (95% CI)**		**198**		**202**	**83.7%**	**1.26 [0.38, 4.18]**
Total events	28		22			
Heterogeneity: Tau² = 0.92; Chi² = 8.55, df = 3 (P = 0.04); I² = 65%						
Test for overall effect: Z = 0.38 (P = 0.71)						
**2.3.2 MDT/ SSDT vs. RDT/ BDT**						
byhahn 2002	3	35	2	35	16.3%	1.55 [0.24, 9.88]
cianchi 2010	0	35	0	35		Not estimable
**Subtotal (95% CI)**		**70**		**70**	**16.3%**	**1.55 [0.24, 9.88]**
Total events	3		2			
Heterogeneity: Not applicable						
Test for overall effect: Z = 0.46 (P = 0.64)						
**Total (95% CI)**		**268**		**272**	**100.0%**	**1.31 [0.50, 3.47]**
Total events	31		24			
Heterogeneity: Tau² = 0.62; Chi² = 8.57, df = 4 (P = 0.07); I² = 53%						
Test for overall effect: Z = 0.55 (P = 0.58)						
Test for subgroup differences: Chi² = 0.03, df = 1 (P = 0.86), I² = 0%						

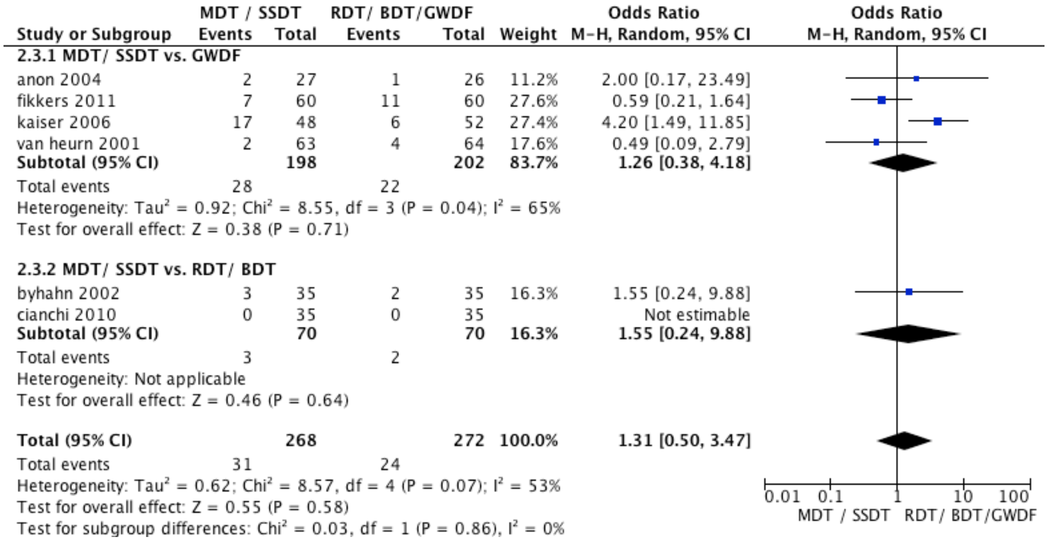

Fig. 2 g) Outcome: False route

Study or Subgroup	MDT / SSDT Events	MDT / SSDT Total	RDT/ BDT/GWDF Events	RDT/ BDT/GWDF Total	Weight	Odds Ratio M-H, Fixed, 95% CI
**2.8.1 MDT/ SSDT vs. GWDF**						
anon 2004	0	27	0	26		Not estimable
fikkers 2011	0	60	1	60	18.6%	0.33 [0.01, 8.21]
nates 2000	0	52	2	48	32.2%	0.18 [0.01, 3.79]
van heurn 2001	0	63	2	64	30.8%	0.20 [0.01, 4.18]
**Subtotal (95% CI)**		**202**		**198**	**81.5%**	**0.22 [0.04, 1.31]**
Total events	0		5			
Heterogeneity: $Chi^2 = 0.08$, df = 2 (P = 0.96); $I^2 = 0\%$						
Test for overall effect: Z = 1.67 (P = 0.10)						
**2.8.2 MDT/ SSDT vs. RDT/ BDT**						
byhahn 2002	0	35	1	35	18.5%	0.32 [0.01, 8.23]
cianchi 2010	0	35	0	35		Not estimable
**Subtotal (95% CI)**		**70**		**70**	**18.5%**	**0.32 [0.01, 8.23]**
Total events	0		1			
Heterogeneity: Not applicable						
Test for overall effect: Z = 0.68 (P = 0.49)						
**Total (95% CI)**		**272**		**268**	**100.0%**	**0.24 [0.05, 1.14]**
Total events	0		6			
Heterogeneity: $Chi^2 = 0.12$, df = 3 (P = 0.99); $I^2 = 0\%$						
Test for overall effect: Z = 1.80 (P = 0.07)						
Test for subgroup differences: $Chi^2 = 0.04$, df = 1 (P = 0.84), $I^2 = 0\%$						

Odds Ratio M-H, Fixed, 95% CI

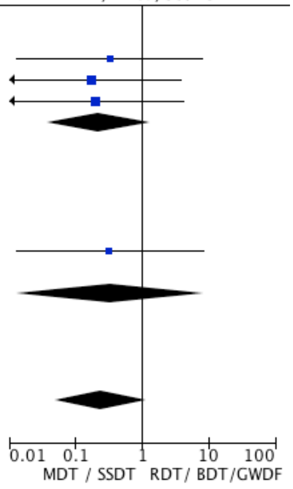

Fig. 2 h) Outcome: Subcutaneous emphysema

Study or Subgroup	MDT / SSDT Events	MDT / SSDT Total	RDT/ BDT/GWDF Events	RDT/ BDT/GWDF Total	Weight	Odds Ratio M-H, Random, 95% CI
**2.5.1 MDT/ SSDT vs. GWDF**						
ambesh 2002	0	30	3	30	35.6%	0.13 [0.01, 2.61]
anon 2004	0	27	0	26		Not estimable
kaiser 2006	1	48	0	52	32.3%	3.32 [0.13, 83.37]
**Subtotal (95% CI)**		**105**		**108**	**67.9%**	**0.62 [0.03, 15.09]**
Total events	1		3			
Heterogeneity: $Tau^2 = 2.78$; $Chi^2 = 2.10$, df = 1 (P = 0.15); $I^2 = 52\%$						
Test for overall effect: Z = 0.29 (P = 0.77)						
**2.5.2 MDT/ SSDT vs. RDT/ BDT/ GWDF**						
byhahn 2002	1	35	0	35	32.1%	3.09 [0.12, 78.41]
**Subtotal (95% CI)**		**35**		**35**	**32.1%**	**3.09 [0.12, 78.41]**
Total events	1		0			
Heterogeneity: Not applicable						
Test for overall effect: Z = 0.68 (P = 0.49)						
**Total (95% CI)**		**140**		**143**	**100.0%**	**1.02 [0.12, 8.92]**
Total events	2		3			
Heterogeneity: $Tau^2 = 1.09$; $Chi^2 = 2.84$, df = 2 (P = 0.24); $I^2 = 30\%$						
Test for overall effect: Z = 0.02 (P = 0.99)						
Test for subgroup differences: $Chi^2 = 0.48$, df = 1 (P = 0.49), $I^2 = 0\%$						

Odds Ratio M-H, Random, 95% CI

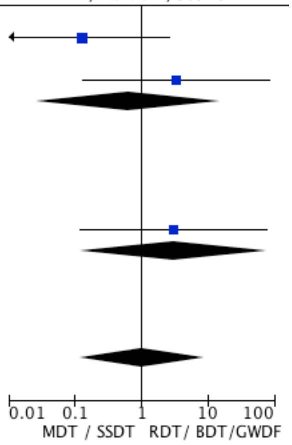

Fig. 2 i) Outcome: Pneumothorax

Study or Subgroup	MDT / SSDT Events	MDT / SSDT Total	RDT/ BDT/GWDF Events	RDT/ BDT/GWDF Total	Weight	Odds Ratio M-H, Fixed, 95% CI
**2.6.1 MDT/ SSDT vs. GWDF**						
ambesh 2002	1	30	0	30	50.5%	3.10 [0.12, 79.23]
anon 2004	0	27	0	26		Not estimable
fikkers 2011	0	60	0	60		Not estimable
kaiser 2006	1	48	0	52	49.5%	3.32 [0.13, 83.37]
nates 2000	0	52	0	48		Not estimable
**Subtotal (95% CI)**		**217**		**216**	**100.0%**	**3.21 [0.33, 31.54]**
Total events	2		0			
Heterogeneity: $Chi^2 = 0.00$, df = 1 (P = 0.98); $I^2 = 0\%$						
Test for overall effect: Z = 1.00 (P = 0.32)						
**2.6.2 MDT/ SSDT vs. RDT/ BDT/ GWDF**						
cianchi 2010	0	35	0	35		Not estimable
**Subtotal (95% CI)**		**35**		**35**		**Not estimable**
Total events	0		0			
Heterogeneity: Not applicable						
Test for overall effect: Not applicable						
**Total (95% CI)**		**252**		**251**	**100.0%**	**3.21 [0.33, 31.54]**
Total events	2		0			
Heterogeneity: $Chi^2 = 0.00$, df = 1 (P = 0.98); $I^2 = 0\%$						
Test for overall effect: Z = 1.00 (P = 0.32)						
Test for subgroup differences: Not applicable						

Odds Ratio M-H, Fixed, 95% CI

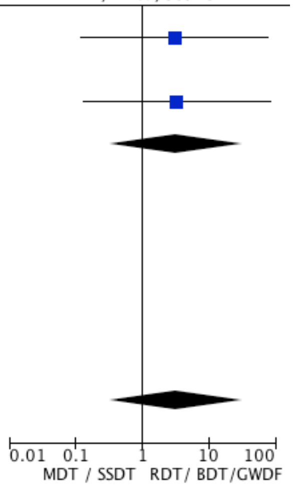

Fig. 2 j) Outcome: Desaturation

Study or Subgroup	MDT / SSDT Events	MDT / SSDT Total	RDT/ BDT/GWDF Events	RDT/ BDT/GWDF Total	Weight	Odds Ratio M-H, Random, 95% CI
**2.4.1 MDT/ SSDT vs. GWDF**						
ambesh 2002	0	30	0	30		Not estimable
anon 2004	0	27	2	26	22.0%	0.18 [0.01, 3.90]
fikkers 2011	1	60	2	60	29.6%	0.49 [0.04, 5.57]
kaiser 2006	5	48	0	52	23.6%	13.28 [0.71, 246.85]
**Subtotal (95% CI)**		**165**		**168**	**75.2%**	**1.04 [0.09, 12.53]**
Total events	6		4			
Heterogeneity: $Tau^2 = 2.79$; $Chi^2 = 4.74$, df = 2 (P = 0.09); $I^2 = 58\%$						
Test for overall effect: Z = 0.03 (P = 0.98)						
**2.4.2 MDT/ SSDT vs. RDT/ BDT/ GWDF**						
byhahn 2002	1	35	1	35	24.8%	1.00 [0.06, 16.65]
cianchi 2010	0	35	0	35		Not estimable
**Subtotal (95% CI)**		**70**		**70**	**24.8%**	**1.00 [0.06, 16.65]**
Total events	1		1			
Heterogeneity: Not applicable						
Test for overall effect: Z = 0.00 (P = 1.00)						
**Total (95% CI)**		**235**		**238**	**100.0%**	**1.02 [0.18, 5.91]**
Total events	7		5			
Heterogeneity: $Tau^2 = 1.17$; $Chi^2 = 4.73$, df = 3 (P = 0.19); $I^2 = 37\%$						
Test for overall effect: Z = 0.02 (P = 0.98)						
Test for subgroup differences: $Chi^2 = 0.00$, df = 1 (P = 0.98), $I^2 = 0\%$						

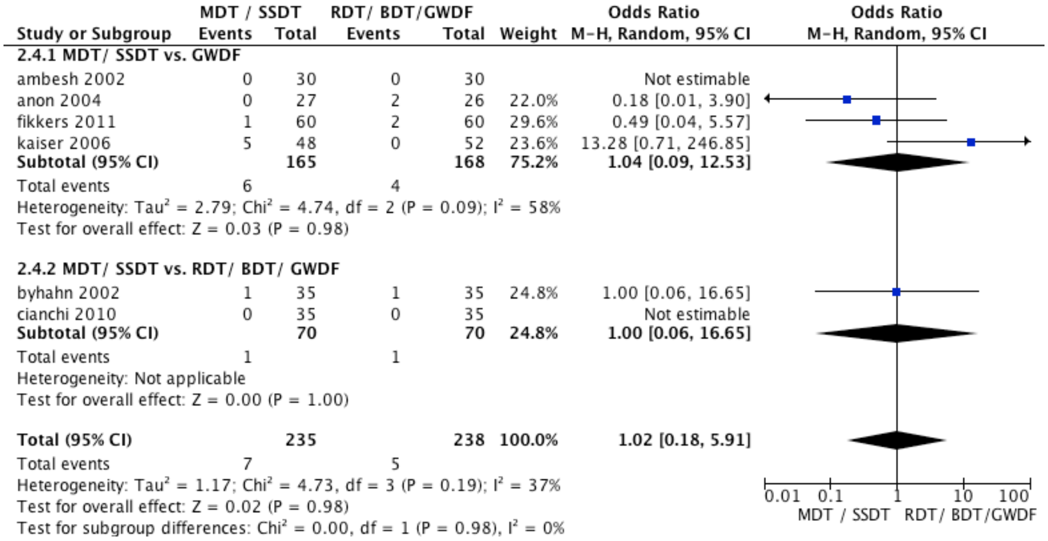

Supplement: Additional file 3: Figures S1 and S2. — Showing the effect of different tracheostomy techniques on complications and outcome – intraprocedural and postprocedural complications. [file 13054_2014_544_MOESM3_ESM.pdf]
